# Cerium-Based Metal–Organic Frameworks: Unveiling the Role of Terahertz Vibrations in the Spin Relaxation Dynamics

**DOI:** 10.1021/acs.inorgchem.4c04542

**Published:** 2025-02-18

**Authors:** Joan Torrent, Cristina Puigjaner, Radovan Herchel, Júlia Mayans

**Affiliations:** † Departament de Química Inorgànica i Orgànica, Secció de Química Inorgànica, 16724 Universitat de Barcelona, Marti i Franques 1-11, Barcelona 08028, Spain; ‡ Departament de Mineralogia, Cristal·lografia i Dipòsits Minerals and Unitat de Difracció de R-X, Centre Científic i Tecnològic de la Universitat de Barcelona (CCiTUB), Universitat de Barcelona, Solé i Sabarís 1-3, Barcelona 08028, Spain; § Department of Inorganic Chemistry, Faculty of Science, Palacký University, Olomouc 77147, Czech Republic; ∥ Institut de Nanociència i Nanotecnologia (IN2UB), Universitat de Barcelona, Barcelona 08028, Spain

## Abstract

The reaction between two equivalents of the Schiff base ligands *N*,*N*′-bis­(3-methoxysalicylidene)­ethylenediamine (H_2_L1) or enantiopure *N*,*N*′-bis­(3-methoxysalicylidene)­cyclohexane-1,2-diamine (H_2_L2) with one equivalent of Ce­(NO_3_)_3_·6H_2_O in the presence of a bulky counteranion leads to the formation of chiral metal–organic frameworks (MOFs) whose channels encapsulate the counteranion, leading to the formation of compounds with the structural formulas {[Ce­(NO_3_)_2_(L1)_2_]­X·H_2_O}_*n*_, where X = ClO_4_^–^ (**1**), PF_6_^–^ (**2**), or BF_4_^–^ (**3**), and {[Ce­(NO_3_)_2_(L2)_2_]­X·CH_3_CN}_*n*_, where X = ClO_4_^–^ (**4**), PF_6_^–^ (**5**), or BF_4_^–^ (**6**), as well as the isostructural reference compound {Nd­(NO_3_)_2_(L1)_2_]­BF_4_·CH_3_CN}_*n*_ (**3Nd**). A combination of static and dynamic magnetic measurements demonstrates the good isolation of the Ce^III^ centers and a field-induced slow relaxation of the magnetization. Correlations between the temperature and field-dependent magnetic relaxation data and ultralow-frequency Raman spectroscopy reveal the presence of a vibronic barrier driving magnetic relaxation. Theoretical calculations have been performed to elucidate the nonparticipation of the electronic excited states in the main relaxation processes.

## Graphical abstract

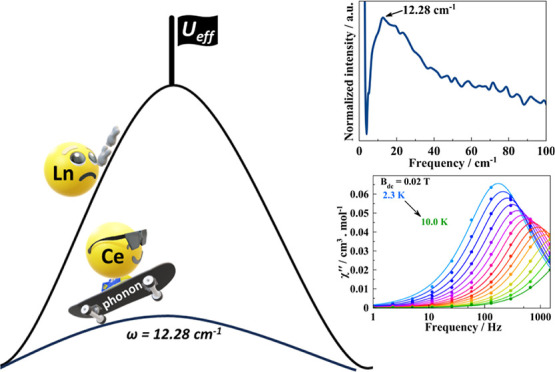

## Introduction

In 1993, the first manifestation of slow relaxation of the magnetization in a single molecule, on the well-known Mn_12_ac,^1^ pioneered extensive research into both unravelling the nature of the mechanisms behind how does such slow relaxation proceed and increasing the performance of these so-called molecular nanomagnets (MNMs). The slow relaxation of these compounds was initially ascribed to the presence of an energy barrier, dependent both on the ground state spin (*S*) and on the axial zero-field splitting (*D*) of the system, the latter being a representation of its magnetic anisotropy. The first approach to accomplish these requirements was to organize first-row transition metal ions (mainly manganese^2,3^ and iron^4,5^ ) into ferromagnetically coupled clusters with large ground spin states. However, as the local anisotropy axes canceled each other out, often these clusters could not retain their magnetization. As a result, the focus was put on maximizing the anisotropy of the system, and lanthanides gained considerable interest. Lanthanides’ high spin–orbit coupling, resulting from the shielding of their 4f orbitals by their 5s and 5p orbitals, leads to an intrinsic single-ion anisotropy, a concept which was proved by Ishikawa’s group when they reported a Tb^III^-based single-ion magnet (SIM) which had a larger anisotropy than any of the first-row transition metal ions.^6^ When designing a lanthanide-based single-molecule magnet, controlling the geometry around the ion is of paramount importance so as to maximize the *D* parameter and minimize the rhombic contribution to reduce quantum tunneling of the magnetization (QTM).^7^ However, the high ionic radii of lanthanides results in highly distorted coordination geometries with elevated coordination numbers, which makes controlling the chemical environment of lanthanide ions a challenging task for synthetic chemists. Taking Rinehardt and Long’s model^8^ model to its limit, in 2017, the liquid nitrogen barrier was broken by a quasi-axial dysprosium metallocene.^9,10^ Although this compound represented a breakthrough in the field, its blocking temperature still remained orders of magnitude below the theoretical barrier. This discrepancy is a consequence of the many under-barrier mechanisms that operate along with the classical Arrhenius-like overbarrier pathway, termed the Orbach relaxation pathway.^11^ Mainly, the one-phonon Direct process and the two-phonon Raman process,^12,13^ along with the well-known QTM, comprise the main relaxation processes of MNMs.

The Orbach process is defined by an exponential dependence; as a result, a small change in the temperature has a large effect on the relaxation rate (τ). However, the Raman process follows a power law, wherein a small change in temperature may not have a such a large effect. Furthermore, in the Raman relaxation pathway, only the ground state is involved, which prevents an involvement with excited states. The Raman relaxation pathway is described in eq 1

1 $$\tau^{-1}=CT^{n}$$

where *C* is taken as a free-fit parameter and the *n* parameter gives valuable information about the nature of the Raman process taking place. Usually, *n* values are 7 and 9 for Kramers and non-Kramers ions, respectively, and 2 for the phonon bottleneck effect.^12^

Recently, some anomalous Raman coefficients have been experimentally found, with *n* values ranging between 3 and 5.^10,14–22^ Two approaches have been taken to justify these deviations: (i) competition between two different relaxation pathways, wherein the resulting *n* value is an average between the two mechanisms, and (ii) the interplay between optical and acoustic phonons involved in magnetic relaxation. The latter approach has been widely used in *S* = 1/2 systems, wherein a large field is usually required to bring about a slow magnetic relaxation with a well-split ground-state doublet. However, in single-molecule magnets with large axial anisotropies, the splitting between the ground-state doublet is usually small enough to disable the energy exchange via a pair of phonons from two different branches. As pointed out in recent publications,^23–25^ these strange Raman coefficients also arise when changing exponential laws to power laws in order to fit through a Raman mechanism. Note that these exponential laws need not imply the presence of an Orbach mechanism but under-barrier processes involving energy barriers from different sources than electronic excited states. One of the main sources of the occurrence of an alternative energy barrier is the hyperfine coupling between the electronic spin and its nuclear spin, leading to an energy barrier formed by the so-called electronuclear states. This is observed in many Co^II^ systems with nonuniaxial anisotropy,^26,27^ where the ground state is *S* = 1/2 and magnetic relaxation has been seen to follow an exponential dependence. In cations without nuclear spin, this relaxation pathway is disabled. However, an Arrhenius-like dependence of magnetic relaxation has been largely observed in one of the most uncommon lanthanoids to exhibit a slow relaxation of magnetization, which does not possess a nuclear spin: Cerium.

Cerium, in its trivalent state, is the first paramagnetic lanthanide in the series. It possesses a single unpaired electron, which, in conjunction with its spin–orbit coupling, leads to a ground state described by the ^2^ *F*_5/2_ term (*S* = 1/2, *L* = 3). Due to its overall low magnetic moment in comparison to that of other lanthanoids such as Dy^III^ (*J* = 15/2), Tb^III^ (*J* = 6), or Er^III^ (*J* = 15/2), it was mostly disregarded to build high-performance SIMs, which is why it was considered as an “uncommon lanthanide”^28^ along with the light lanthanides Pr^III^, Nd^III^, and Sm^III^, the isotropic Gd^III^, and Ho^III^, Tm^III^, and Yb^III^. However, a literature search reveals that in any of the reported slowly relaxing monometallic Ce^III^-based systems, such a barrier plays a role.^19,20,29–35^ With the experimentally determined barrier being consistently 10-fold lower than the calculated one, even in the zero-field slowly relaxing Ce^III^ system,^29^ this small energy barrier was found to be the consequence of the relaxation of the magnetization through local vibrational modes, with the operating phonons being purely from the optical branch, in a relaxation pathway coined local modes, with a small energy barrier termed the vibronic barrier. As a result, Ce^III^-based systems are an interesting playground from which to investigate these relatively nonconventional relaxation pathways. Due to the lack of electronuclear states and, following the literature, the lack of interaction between the electronic excited states, Ce^III^-based systems may be considered as effective *S* = 1/2 systems, which entail an easy manipulation of the electronic states.

In recent years, effective *S* = 1/2 systems have found potential applications in the field of quantum information processing,^16,17,36^ which has granted Ce^III^ a considerable spike in interest. As of today, the number of reported ceria-based SIMs remains extremely low. And, to the best of our knowledge, only two are based on metal–organic frameworks (MOFs).^37,38^ MOFs have found applications in areas such as catalysis,^39–41^ molecular recognition,^42,43^ sensing,^44,45^ or gas capture and storage.^46,47^ They can be assembled through the linkage of metal nodes with organic linkers. Moreover, the integration of spin carriers into these frameworks grants spatial control over the spin–spin distance, which in turn grants control over interspin interactions.^48^ Such control is key to fulfilling these systems’ aforementioned potential applications.^18,49–52^

With the purpose of constructing Ce­(III)-based MOFs and given our previous experience,^53–55^ we have chosen Schiff bases as easily tunable ligands: their versatility, polydenticity, and π-rigidity around the donor atoms afforded by the Schiff bases used provide an optimal environment from which to build multidimensional MNMs.

Specifically, we chose the hexadentate Schiff bases derived from the condensation reactions of o-vanillin with either ethylenediamine (**H**_**2**_ **L1**) or the enantiopure (1*R*/*S*, 2*R*/*S*)-1,2-cyclohexanediamine (**H**_**2**_ **L2**) (Chart 1). These ligands typically contain two cavities in their bis-tetradentate usual coordination mode: the two iminic nitrogens and the phenolic oxygens comprise a small cavity, which is usually employed to hold a 3d cation. The outer cavity is composed of the phenoxo and methoxo units and is used to hold 4f cations. Commonly, these ligands are employed as two-ion holding hexadentate ligands, which is why in the literature, one can find many 3d–4f multinuclear systems^56–61^ based on these ligands, which have been proposed for many different applications, given their multifunctionality with properties such as SMM behavior,^61–63^ luminescence,^64^ or even catalytic activity.^65,66^ However, the nature of the ligand enables the torsion of the diamine and, as a result, its usage as a bridging bis-monodentate ligand, thus disabling its typical coordination mode (Chart 1). Through the latter coordination motif and the addition of a suitable counteranion, such as ClO_4_^–^, PF_6_^–^, or BF_4_^–^, we achieved compounds with the structural formulas {[Ce­(L1)_2_(NO_3_)_2_]­(ClO_4_)·CH_3_CN}_*n*_ (**1**), {[Ce­(L1)_2_(NO_3_)_2_]­(PF_6_)·CH_3_CN}_*n*_ (**2**), {[Ce­(L1)_2_(NO_3_)_2_]­(BF_4_)·CH_3_CN}_*n*_ (**3**), {[Nd­(L1)_2_(NO_3_)_2_]­(BF_4_)·CH_3_CN}_*n*_ (**3Nd**), {[Ce­(L2)_2_(NO_3_)_2_]­(ClO_4_)·CH_3_CN}_*n*_ (**4**), {[Ce­(L2)_2_(NO_3_)_2_]­(PF_6_)·CH_3_CN}_*n*_ (**5**), and {[Ce­(L2)_2_(NO_3_)_2_]­(BF_4_)·CH_3_CN}_*n*_ (**6**) and the magnetically diluted complex **1Ce20%@La**. The structures of reference are the neodymium analogue **3Nd** and the previously reported^67^ complex **5**. The obtained compounds were found to be isostructural across counteranions, that is, **1**–**3** and **4**–**6**, as probed by powder X-ray diffraction (PXRD). The magnetic behavior of compounds **1** and **4** was interrogated through SQUID magnetometry. These MOFs present slow relaxation of the magnetization under a small applied magnetic field with very similar responses in between them.

**Chart 1:**
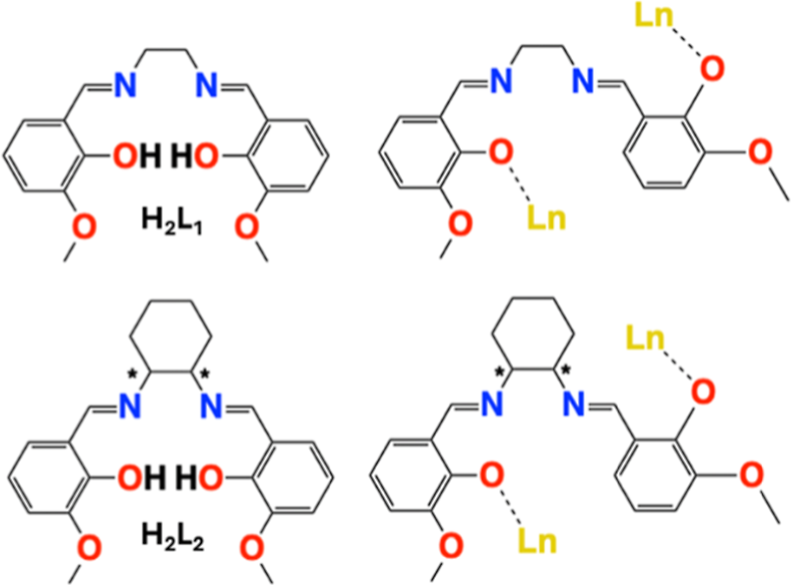
Top and Bottom Left: Ligands Used in This Work, the Asterisks Denote Chiral Centers; Top and Bottom Right: Schematical Representation of the Coordination Mode of the Ligands to the Lanthanide Cations

The field-dependent behavior of compound **1** was studied in depth and correlated with its ultralow-frequency Raman spectrum. The magnetic response was also studied in a Ce-doped diamagnetic La^III^ analogue (**1Ce20%@La**). Furthermore, ab initio theoretical calculations were performed to support the nonparticipation of the electronic excited states in magnetic relaxation, leading to the observation of a remarkably large energy barrier and the observation of a semicoordination between the nitrato ligands and the Ce^III^ ion, which might be the cause of such a large energy barrier.

## Experimental Section

### Materials, Methods, and Physical Measurements

All reagents and solvents were used as received with no further purification. Infrared spectra were recorded in the 4000–400 cm^–1^ range on a Thermo Nicolet scientific IS5 spectrophotometer, with KBr as a dispersing agent and the OMNIC software package for visualization of the spectra. Magnetic susceptibility and magnetization measurements were performed on pressed polycrystalline samples of **1** and **4** in the 2–80 K range with a Quantum Design MPMS-XL SQUID magnetometer at the CCiT Magnetochemistry Unit of the University of Barcelona. Diamagnetic corrections were estimated from Pascal tables. Ultralow-frequency Raman spectra were recorded with a high-resolution Raman T64000 (Jovin Yvon) instrument.

### X-ray Crystallography

Single crystal X-ray diffraction (SC-XRD) was performed on the reference compound **3Nd** (Table S1, Figures S1–S3). Yellow block-like specimens were mounted on a Bruker D8 Venture system equipped with a multilayer monochromator and a Mo microfocus (λ = 0.71073 Å). The frames were integrated with the Bruker SAINT software package using a narrow-frame algorithm. The structure was solved and refined using the Bruker SHELXTL Software Package.^68^ PXRD was performed on a PANalytical X’Pert PRO MPD θ/θ powder diffractometer of 240 mm radius, in a configuration of convergent beam with a focalizing mirror and a transmission geometry with flat samples sandwiched between low absorbing films. Cu Kα radiation (λ = 1.5418 Å) was employed with a work power of 45 kV to 40 mA. The scans were performed in the 2–60° 2θ range with a step size of 0.0263° 2θ and a measuring time of 300 s per step and refined using the Bruker SHELXTL software.

### Computational Details

The theoretical calculations were done with the ORCA 6.0 software.^68,69^ The Douglas–Kroll–Hess (DKH) Hamiltonian was applied to treat relativistic effects,^70,71^ together with SARC2-DKH-QZVP for lanthanide atoms and DKH-def2-TZVP for all other atoms except for C and H atoms, for which the DKH-def2-SVP basis was used.^72,73^ The calculations were sped-up using the SARC/J coulomb fitting basis set^74^ and RIJCOSX approximation.^75–77^ The largest integration grid (DefGrid3) was used in all of the calculations. The molecular structures were extracted from X-ray data, and the atomic positions of hydrogen atoms were optimized with the BP86 DFT functional together with atom-pairwise dispersion correction (D4).^78^ The subsequent DFT calculations were done with the help of the ωb97x-V range-separated hybrid funcional,^78^ and the calculated electron density was analyzed with AIMAII software.^79^ Next, the state average complete active self-consistent field (SA-CASSCF)^80^ wave function method was employed for selected complexes, and in the case of Ce^III^ complexes, these calculations were complemented by N-electron valence second-order perturbation (NEVPT2).^81,82^ The active space was defined by seven f-orbitals, and numbers of multiplets were determined from the respective electronic configuration; in the case of the Ce^III^ ion with 4f^1^, 7 doublets were calculated, and for the Nd^III^ ion with 4f^3^, 35 quartets and 112 doublets were calculated. The SINGLE_ANISO module^83^ was also employed. The calculated data were visualized with the Diamond 5 program.^84^

### Syntheses

The syntheses of the ligands have been carried out following a previously reported procedure.^67^

#### H_2_L1

To a stirred solution of ethylenediamine (1 mmol, 0.060 g) in 10 mL of methanol, *o*-vanillin (2 mmol, 0.300 g) was added. The yellow solution was allowed to stir for 1 h at room temperature, after which a yellow precipitate was observed. The solid was filtered and dried under vacuum to obtain a yellow powder in almost quantitative yield. IR (KBr, cm^–1^): 3432 (m), 2930 (w), 1632 (s), 1468 (s), 1248 (vs), 1080 (ms), 962 (ms), 730 (m).

#### H_2_L2

To a stirred solution of (*R*,*R*)-1,2-diaminocyclohexane (0.2 mmol, 0.022 g) in 10 mL of a 1:1 methanol/acetonitrile mixture, *o*-vanillin (0.4 mmol, 0.060 g) was added. The dark yellow solution was allowed to stir for 1 h at room temperature, during which no precipitate was obtained. The resulting yellow solution was employed in situ to perform the syntheses of the frameworks.

#### Metal–Organic Frameworks

The frameworks were synthesized by adapting a previously reported procedure.^67^ To a stirred solution of 0.2 mmol corresponding ligand in 10 mL of a 1:1 methanol/acetonitrile solution, Ce­(NO_3_)_3_·6H_2_O (0.1 mmol, 0.066 g) was added. Upon the addition of the cerium salt, the bright yellow solution acquired a darker color. After stirring the solution for 2 h, the corresponding anion was added in the form of its tetramethylammonium (TMA) salt: TMA­(ClO_4_) (0.3 mmol, 0.053 g), TMA­(PF_6_) (0.3 mmol, 0.065 g), or TMA­(BF_4_) (0.3 mmol, 0.048 g). The solution was allowed to stir overnight, and a mustard yellow precipitate was collected by filtration and dried in vacuo. Single crystals of the slightly soluble **3Nd** analogue were obtained by leaving the mother liquor unperturbed for 1 week. The magnetically diluted **1Ce20%@La** was obtained following the same procedure starting from a mixture of La­(NO_3_)_3_·6H_2_O and Ce­(NO_3_)_3_·6H_2_O in a 4:1 ratio.

The IR and powder X-ray spectra of all the prepared compounds are shown in Figures S4 and S5, respectively.

## Results and Discussion

### Description of the Structures

Detailed description of the structure of complex **5** can be found in ref 67. The following description of the structure of **3Nd** is compared with the main features of **5** to remark the similar arrangement and coordination environment and any significant differences prompted by the more rigid and chiral diamine 1,2-cyclohexanediamine (which yields compounds **4**–**6**, also isostructural among them).

Partially labeled plots of **3Nd** and **5** are shown in Figure 1. Their selected bond distances, angles, and torsions are listed in Table S2. Structures **3Nd** and **5** show neutral three-dimensional MOFs, which crystallize in the *P*6_2_22 and *P*6_4_22 space groups, respectively. Regarding the first coordination sphere around Nd^III^, each cation is coordinated to two bidentate nitrato ligands and four monodentate L^2–^ ligand units through their phenolato donor, thus achieving an overall coordination number of 8. No solvents are coordinated to the cation, and acetonitrile molecules were found to be present in the lattice, evaporation of which did not cause a change in such lattice. Continuous shape measurements (CShM) performed with the SHAPE software^85^ show extremely distorted geometries for both compounds, with the lowest CShM coefficients being 7.72 and 7.88 for **3Nd** and **5**, respectively, for hexagonal bipyramidal geometries (Figure S6). Such strong distortion might arise from the low bite angles of the nitrato ligands. The Nd–O distance is slightly larger for the phenolato than for the nitrato ligands by 0.9 and 0.6 Å for **3Nd** and **5**, respectively, which also heavily contributes to the increase in the distortion from the ideal hexagonal bipyramid. Since each ligand bridges two metal centers and each metal center is coordinated to four monodentate L^2–^ units (Figure 1), each cation acts as a node, being bridged to four other nodes. The Nd–Nd distance is of 10.285 Å, indicating an optimal isolation of the spin carriers. It is noteworthy that the intercation distance is slightly larger in **5** than in **3Nd** (9.828 Å). Moreover, the Ln···Ln···Ln angle is considerably smaller in **5** than in **3Nd**, with angles of 101.90 and 111.79°, respectively (Table S2) attributable to the flexibility of the aliphatic fragment. As for the lattice extension, the bridge-like role of the ligands along with the aforementioned coordination motif brings about a helical-like metal–ligand–metal extension along the *c* crystallographic axis (Figure 2a,c). Chiral helices with both the enantiopure H_2_L2 and the nonchiral H_2_L1 ligands were obtained. The helicity in both of our examined crystals was found to be right-handed. Chargewise, the two negative charges resulting from the deprotonation of the phenoxo units when they become coordinated to the cation are compensated through the protonation of the two iminic nitrogens. The two nitrato ligands are not enough to offset the +3 charge on the Ln^III^ cation, which leaves a +1 net charge, which is balanced out by the presence of the monoanionic counteranion. The counteranions are located in hexagonal channels formed by 6 helices (Figure 2b,d), wherein each helix acts as a vertex of the hexagon. It is also worth pointing out that the channel widths (measured as the distance between hexagon vertices) are 11.610 and 8.481 Å for **3Nd** and **5**, respectively.

**Figure 1:**
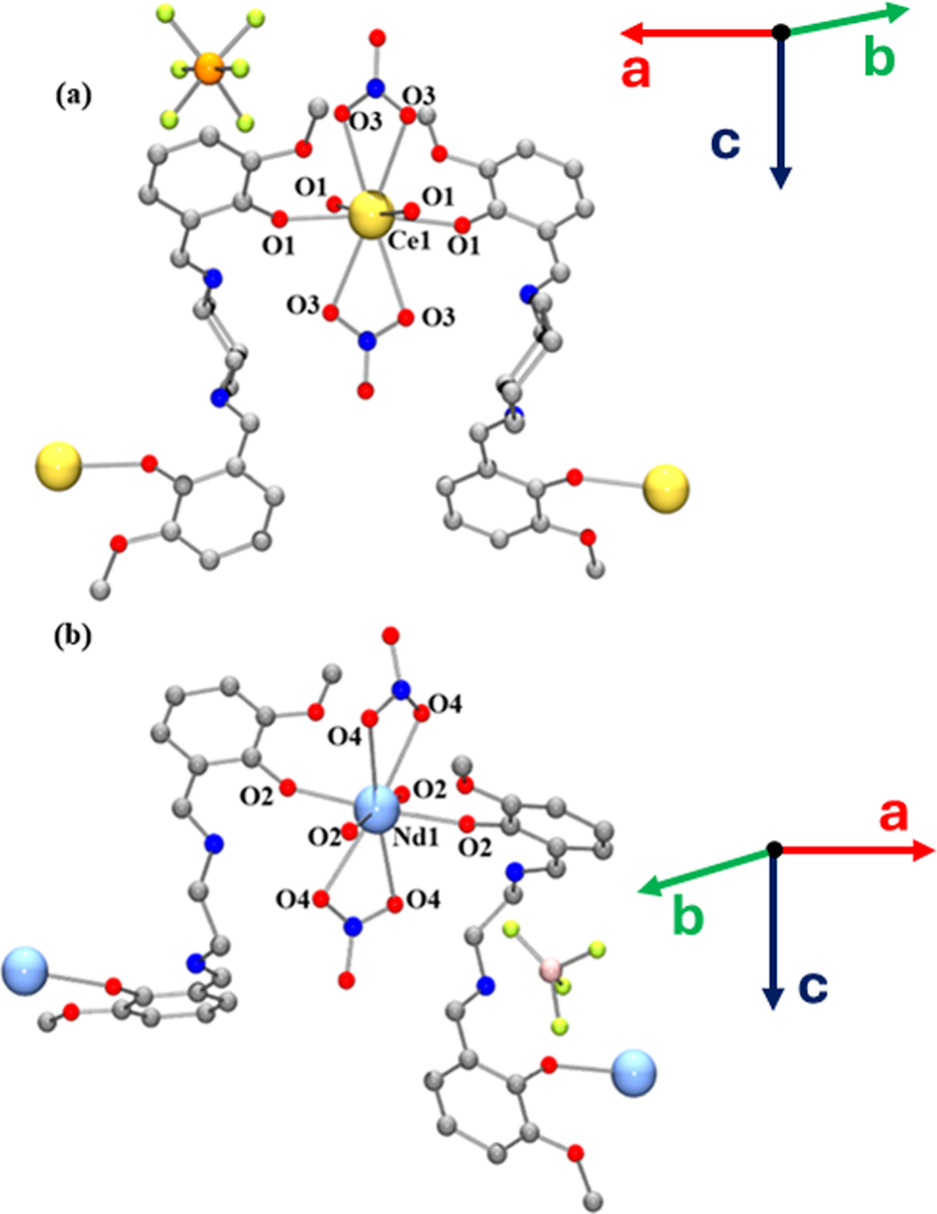
Partially labeled plots of compounds **5** (a) and **3Nd** (b). Data for complex **5** from CCDC (FEZQEI).

**Figure 2:**
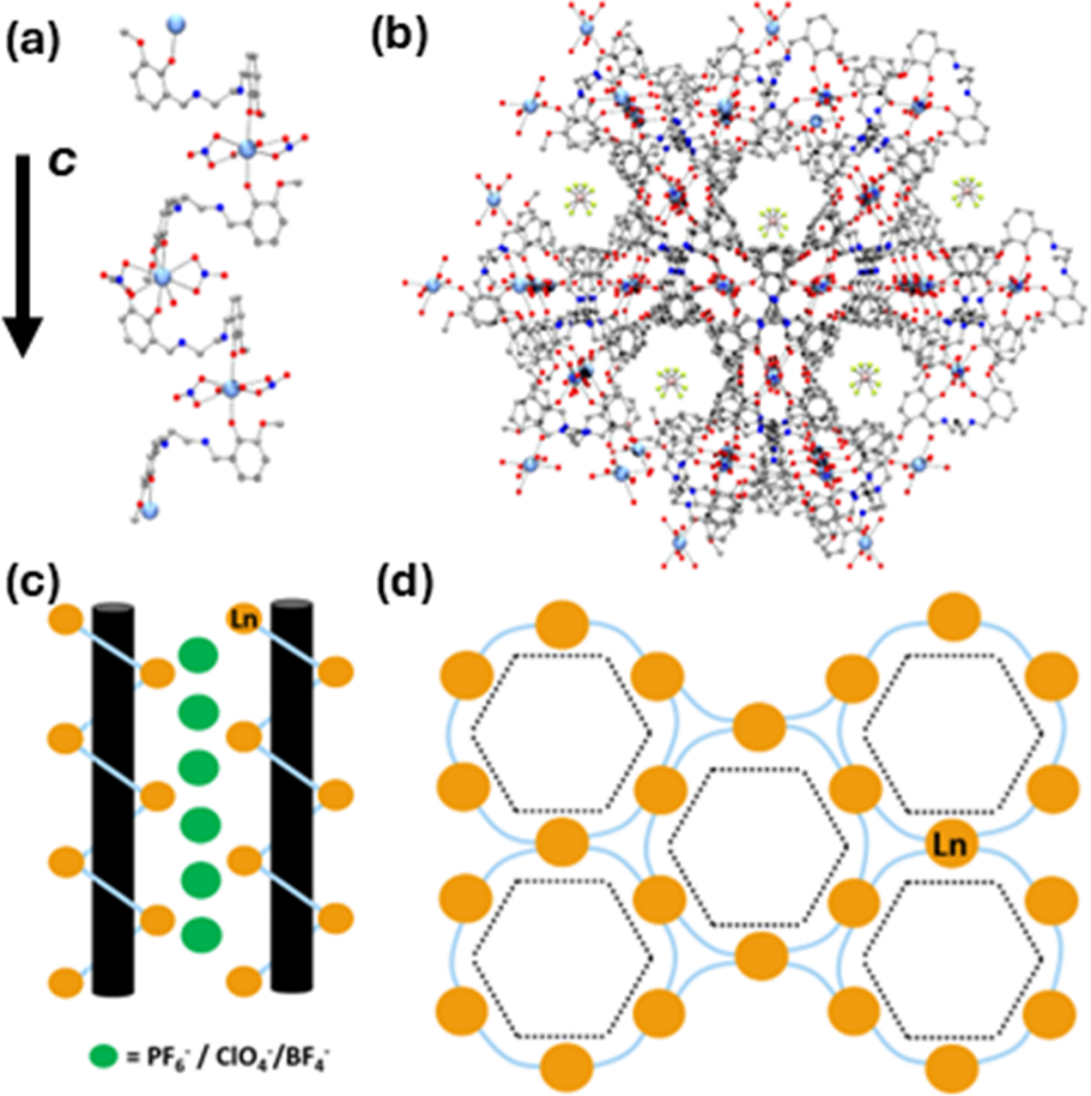
Plot (a) and schematical representation (c) of the helical-like arrangement down the c crystallographic axis of **3Nd**. View (b) and schematical representation (d) of **3Nd** along the c crystallographic axis (across the *ab* plane).

### Magnetic Properties

#### Static Measurements

Preliminary static and dynamic measurements for the complexes derived from H_2_L1 (systems **1**–**3**) and H_2_L2 (systems **4**–**6**) were exactly superimposable inside each series, and thus, the detailed magnetic properties have been only studied for the perchlorate compounds **1** (Ce^III^ and L1^2–^) and **4** (Ce^III^ and L2^2–^).

Magnetic susceptibility was recorded as a χ_M_ *T* product versus temperature in the 2–80 K temperature range (Figure S7a) for compounds **1** and **4**. Upon cooling, the χ_M_ *T* values decrease until reaching values of 0.42 and 0.44 cm^3^ mol^–1^ K at 2 K for compounds **1** and **4**, respectively. Such a decrease is a consequence of depopulation of the thermally excited *M*_J_ (or Stark) sublevels. It is noteworthy that the experimental data obtained in the magnetic susceptibility measurements nicely agree with the results from the CASSCF calculations (Figure S21). The magnetization curve was recorded at 5 K (Figure S7a, inset). Upon increasing the magnetic field, the magnetization starts to increase until reaching a saturation value of 1 Nμ_B_, corresponding to a ^2^F_5/2_ with a *g* value of 6/7, at about 6 T; for compound **1**, the magnetization is not saturated at 5 T, which is commonly used as an indicator of high anisotropy. For **1**, another factor that supports the high anisotropy of the system is the reduced magnetization plots (Figure S7), which show superimposable curves, the consequence of the large *D* of the spin carriers in this framework. Such a large anisotropy was also verified through ab initio calculations, wherein an energy splitting between the ground and first excited states close to 700 cm^–1^ was found (see below).

#### Dynamic Measurements

Preliminary alternate current (AC) measurements for compounds **1**, **3Nd**, and **4** reveal that neither has an out-of-phase response at zero-field, which can be attributed to a fast and effective QTM, common in largely distorted environments of the spin carriers due to the presence of some degree of transverse anisotropy (*E*). Under the application of a small static field, QTM gets progressively quenched for **1** and **4**, and out-of-phase signals arise for both compounds, while there is no response for **3Nd**, as is shown in the field-sweep measurements in Figure S8. Compound **1** shows weak tails at the smallest studied fields (0.002–0.01 T), whereas from 0.02 T onward, clear out-of-phase maxima can already be seen (Figure S8, left). In the diluted analogue **1Ce20%@La**, these maxima already appear at fields of 0.002 T (Figure S9). The position of these maxima moves to higher or lower temperatures at the highest fields (0.7–1.2 T) and at the lowest fields (0.02–0.03 T) fields, respectively, while it is field invariant in the measured intermediate fields (from 0.3 to 0.7 T) (Figure S8). In the high field range, the position of the maxima shifts toward lower temperatures, which may indicate that the direct mechanism has started to kick in. To further study the relaxation dynamics of **1**, we selected the field of 0.02 T in the 10–1488 Hz frequency range and 2–10 K temperature range (Figure S10).

For compound **4**, neither the positions nor the intensities of the out-of-phase maxima change with the magnitude of the applied field (Figure S8, right). Like in compound **1**, we selected an optimal field of 0.02 T to study the temperature dependence of the out-of-phase response in the 10–1488 Hz frequency range and in the 2–8 K temperature range (Figure S10).

The obtained frequency-dependent data for both compounds were fitted through the generalized Debye model^86^ (Figure 3) using self-made software. From a preliminary ln­(τ) vs ln­(*T*) plot, otherwise known as log–log plots (Figure S12), there are two clear and differentiated slopes for both compounds, which elucidates the presence of two distinct relaxation mechanisms, each dominating in a specific range of temperatures. This observation is further confirmed by the evolution of the alpha parameter across the studied temperatures, oscillating from 0.22 at low temperatures to 0.05 for compound **1** and from 0.3 to 0.01 for compound **4** at high temperatures (Figure S11) and indicating a broad-to-narrow distribution of the relaxation times. The fitted data were further represented in the form of ln­(τ) vs the inverse of the temperature in an Arrhenius plot for **1** and **4** (Figure 4). The data could be fitted to many different combinations of relaxation pathways, which are summarized by the Debye equation (eq 2)­

2 $$\tau_{1}^{-1}=CT^{n}+QTM+\tau_{0}^{-1}e^{-E_{a}/KT}$$

where the first, second, and third terms correspond to Raman, QTM, and an exponential law-following relaxation pathway, respectively. Note that, in any case, the latter term can correspond to an Orbach-like relaxation pathway due to (i) the remarkably large zero-field splitting values obtained from the ab initio calculations (see below) and (ii) the behavior of the maxima in the χ_M_^″^ vs frequency in a variable magnetic field and constant temperature representation (Figure 5left, center; S16). The obtained values from the different combinations of relaxation mechanisms for **1** and **4** are summarized in Table S3. For **1** and **4**, different fitting options were considered. Two of them involve direct relaxation, which, given the DC field at which the AC data were taken, should not be effective enough to drive magnetic relaxation, whereas the other possibility involves a combination of Raman and QTM. This combination of the fitting for both compounds agrees with the shapes of the curves in Figure 4: at high temperatures, the curve follows the straight line that is usually related to an Orbach-like relaxation (dotted line), and when the temperatures are lowered, the shape of the curve starts to follow the Raman-like power law function. It has to be realized at this point that in the Orbach-like regime, a small reduction in temperature can dramatically increase the relaxation time (see above), while when the Raman-like regime is reached, the sensitivity of relaxation time with temperature gets weakened because of the transition to the power law. Finally, at very low temperatures, the curve arrives at a temperature-independent regime, corresponding to the QTM relaxation. He obtained Raman coefficients in a pure Raman + QTM fit stand out as they are highly deviated from the usual *n* values for Kramers ions or for the phonon-bottleneck effect (which is discarded to participate because there is no worsening of the AC measurements at the same field in the magnetically diluted samples of the studied compounds **1Ce20%@La** (Figure S9)).^87,88^ Such deviations from the theoretical Raman coefficients have been reported and explained before (see above). However, in **1** and **4**, due to the presence of a high degree of axial anisotropy, the presence of phonons of different types is highly unlikely.^23^ Relaxation driven by purely optical phonons (i.e., intramolecular vibrations or local vibrational modes) can lead to exponential ln­(τ) vs *T* ^–1^ dependencies due to the presence of the so-called vibronic barriers.^24,25^ With the goal of obtaining the experimental value of this vibronic barrier, the last term of eq 3 was used. And the obtained effective energy barrier values from considering an exponential law were 8.60 and 8.91 cm^–1^ for compounds **1** and **4**, respectively.

**Figure 3:**
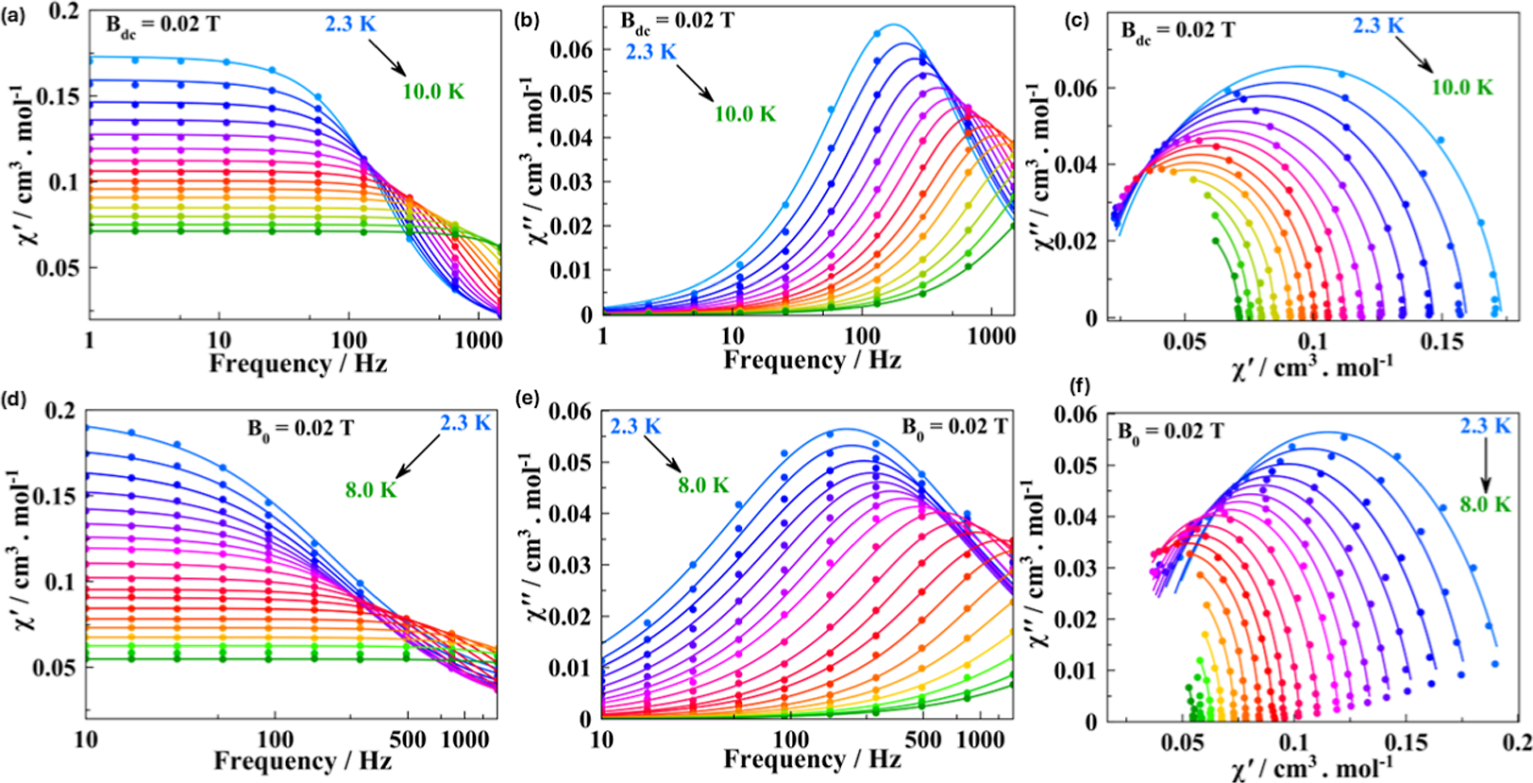
In-phase (a,d), out-of-phase (b,e), and Cole–Cole (c,f) plots for compounds **4** and **1**, respectively, recorded at a fixed static field of 0.02 T.

**Figure 4:**
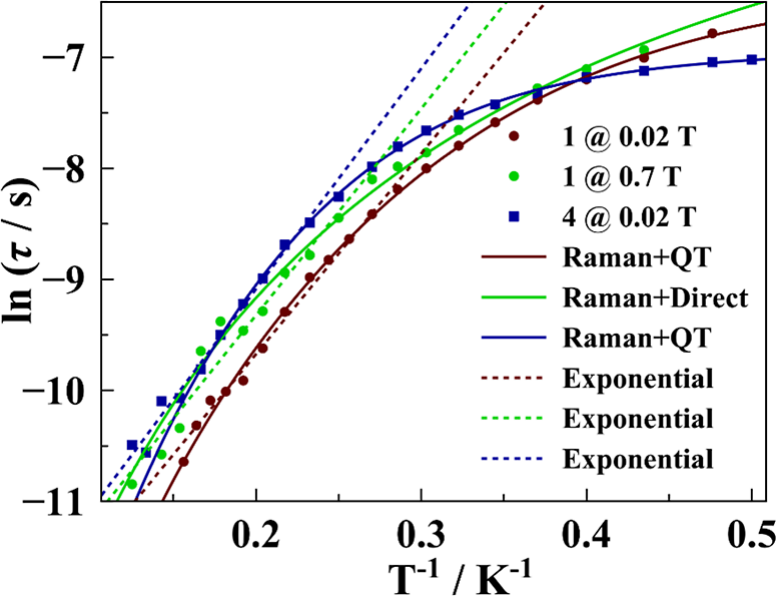
Plots of ln­(τ) vs the inverse of the temperature for complexes **1 (d–f)** and **4** (a–c). Solid lines correspond to the best fits. Dashed lines correspond to the exponential section of the curve.

**Figure 5:**
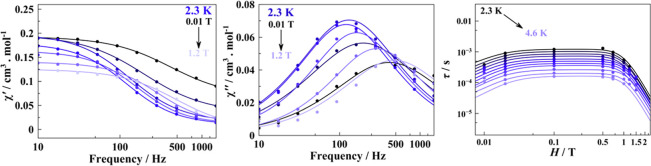
In-phase (left) and out-of-phase (center) components of the magnetic susceptibility recorded at variable applied dc fields at a fixed temperature of 2.3 K for complex **1**. Right, relaxation time dependence on the applied static field in the 2.3–4.6 temperature range; solid lines indicate the best fits.

The presence of exponential regimes in Raman-like relaxation is ascribed to the presence of phonons purely from the optical branch. These optical phonons represent local vibrational models (i.e., intramolecular vibrations) rather than the lattice vibrations generated by acoustic phonons. Unlike the Raman relaxation process, the local modes pathway involves well-defined vibrational energy levels. As a result, we pursued an alternative approach to fit the AC data, wherein we replace the Raman term with the local modes term (eq 3)­

3 $$\tau_{1}^{-1}=QTM+D\left(\frac{e^{-\omega /k_{B}T}}{{\left(e^{-\omega /k_{B}T}-1\right)}^{2}}\right)$$

where the first and second terms correspond to QTM and local modes relaxation pathways, respectively, and ω is the energy of the vibrational mode associated with the latter pathway or, in other words, the vibrational mode from which the so-called vibronic barrier arises. The obtained values in these fits are also summarized in Table S3, with ω values of 12.64 and 13.46 cm^–1^ for compounds **1** and **4**, respectively.

To gain further insights into the relaxation dynamics of these MOFs, we performed an extensive analysis on compound **1**, where we studied the evolution of its relaxation dynamics as a function of the applied DC field in the 2.3–8 K temperature range and the 0.01–1.2 T field range.

The experimental τ vs *H* values are represented in Figure 5 as isothermal scatters. From Figure 5, three different sections can be easily distinguished: first, an increase in τ is seen from 0.01 to 0.1 T (Figures S16–S18) due to the suppression of QTM by the increasingly applied field. The main source of QTM in these compounds was found to be the spin–spin dipolar interactions, which were partially suppressed upon magnetically diluting Ce^III^ in an isostructural La^III^ diamagnetic analogue **1Ce20%@La** (Figure S9). This magnetic dilution led to clear maxima appearing at fields lower than 200 G (Figure S9), in contrast to the nondiluted compound **1**. The second section of Figure 5 (from 0.1 to 0.7 T) features a practically invariant τ vs *H* dependence (Figures S16–S18). In this field range, QTM is effectively quenched, and the local modes process (which is a second-order Raman process) dominates the magnetic relaxation, along with a small but quasi-constant contribution from the direct process. When the field surpasses 0.7 T (and up to 1.2 T), the direct process takes over, which is characterized by the *H* ^4^ dependence of the relaxation rate, which results in a rapid decrease in the relaxation time and, ultimately, the vanishing of the AC response at fields higher than 1.2 T (Figures S16–S18).

Considering the aforementioned relaxation pathways, the field-dependent data were fit to eq 4

4 $$\tau_{1}^{-1}=\frac{1+B_{1}}{1+B_{2}H^{2}}+d\left(\frac{1+eH^{2}}{1+fH^{2}}\right)+cH^{4}$$

where the first, second, and third terms correspond to QTM, field-dependent Raman (otherwise known as the Brons–Van Vleck equation), and direct pathways, respectively. The obtained fits at each temperature are shown in Figure S18, and the obtained parameters are given in Table S5. Among eq 4, we put our focus on the *d* parameter, which represents the zero-field relaxation. This parameter was found to possess an exponential dependence on the temperature along the studied 2.3–4.6 K range. Noteworthily, such exponential dependence is not related to an Orbach-like relaxation, rather it indicates that there are well-defined energy levels involved in spin–phonon relaxation.^17^ Given the nature of the relaxation processes taking place, these energy levels might correspond to the vibrational levels of the molecule. The magnitude of the energy level at play can be determined through

5 $$\ln\left(d\right)=\ln\left(a\right)-\frac{U_{eff}}{K_{B}T}$$

This relationship is confirmed in Figure 6, inset, and in Figure S19, which yields an energy barrier of 9.24(4) cm^–1^. Upon introducing the obtained value for the energy barrier into eq 6 ^17^

6 $$U_{eff}=\frac{h\omega }{2}$$

**Figure 6:**
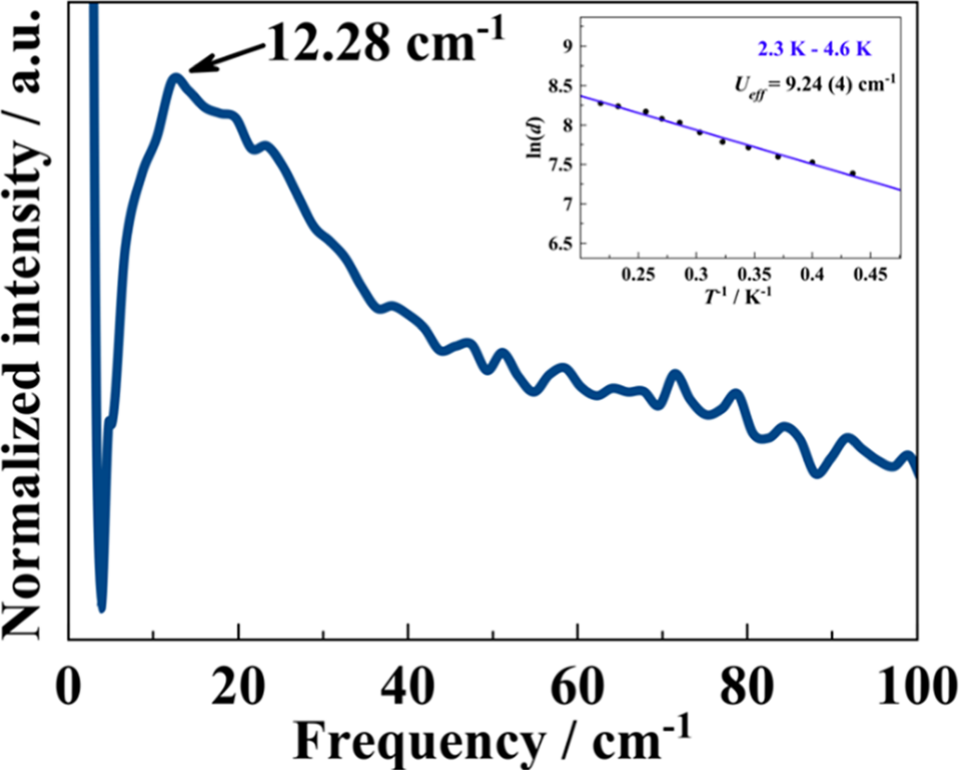
Ultralow-frequency Raman spectrum of **1**. Inset, ln­(*d*) vs the inverse of the temperature relationship; the solid line corresponds to a linear fit in the 2.3–4.6 K temperature range.

We found that the obtained energy barrier comes from a vibrational mode of 12.84 cm^–1^. To further probe the presence of these low-energy phonons, we performed ultralow-frequency Raman spectroscopy on a polycrystalline sample of **1** in the 0–100 cm^–1^ frequency range (THz region) at room temperature (Figure 6), where we found different absorption peaks at 12.28, 19.70, and 23.40 cm^–1^. The first of these was found to be in perfect correlation with the vibrational mode found at 12.84 cm^–1^ through applying the Brons–Van Vleck equation (eq 4, middle term) and to the fitted local mode value (Figure 6 and Table S3) of 12.64 cm^–1^. Furthermore, the obtained activation energy of 9.24 cm^–1^ is also in good agreement with the obtained value of 8.60 cm^–1^ from fitting our AC data to an exponential law. This work represents one of the first experimental evidence of the presence of vibronic barriers involved in a slow magnetic relaxation process and of its experimental determination through Raman spectroscopic measurements. Furthermore, this is the first time the quadruple correlation between (i) the pure exponential fit, (ii) the value extracted from the fitting to a local modes process, (iii) the vibrational mode obtained through the Brons–Van Vleck equation, and (iv) experimental spectroscopic data has been performed.

### Computational Results

ORCA 6.0 was applied to treat the electronic and magnetic properties in selected complexes **3Nd** and **5Ce**. Due to the polymeric character of these MOFs, the treatable molecular fragments (Figure 7) were extracted from experimental XRD data of these compounds (Figures 1, 2, and S1–S3).

**Figure 7:**
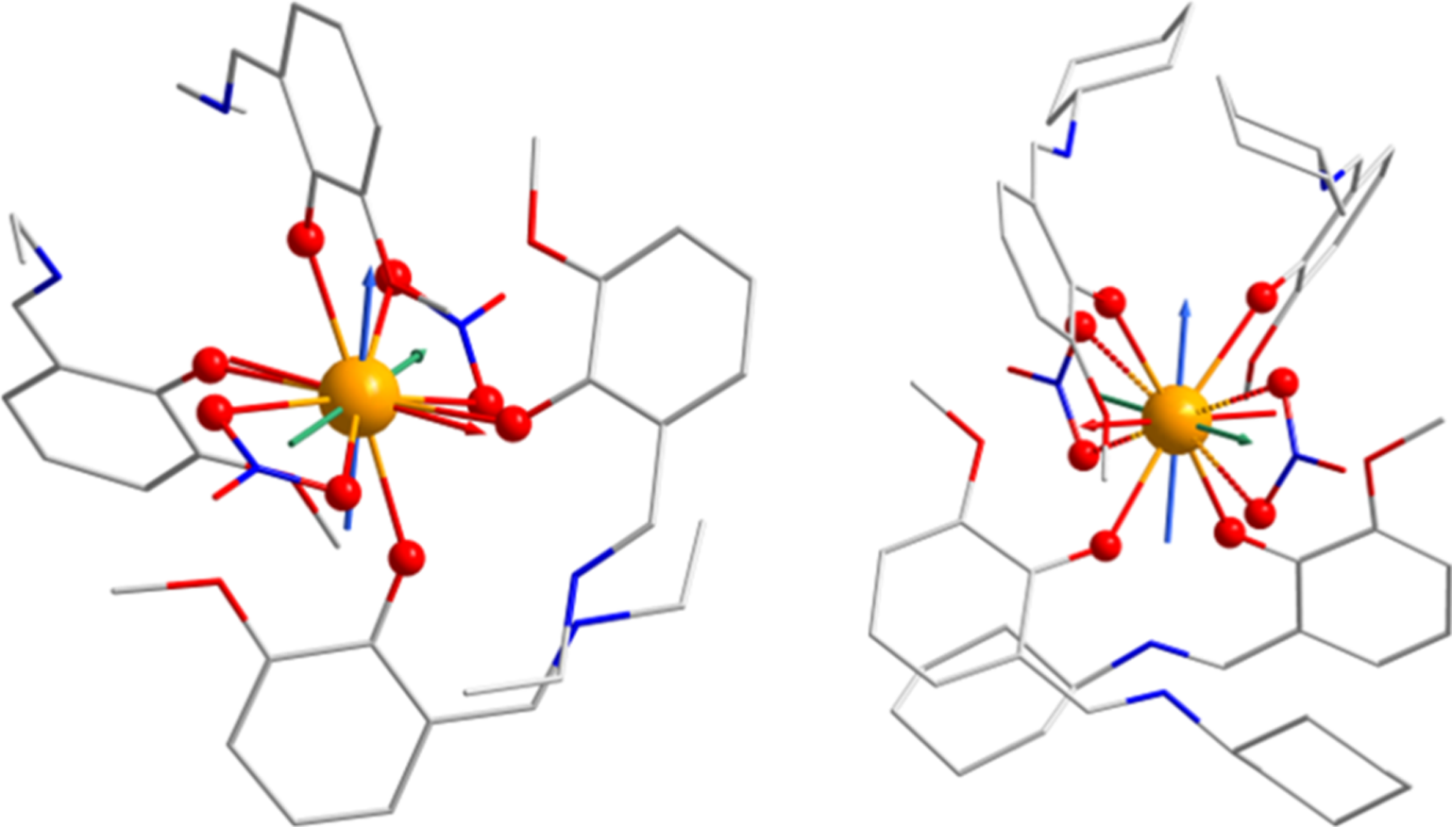
Molecular fragments of **3Ce**′ (left) and **5Ce** (right) used for theoretical calculations overlaid with the principal axes of ground-state Kramers’ doublet *g*-tensor (*x*/*y*/*z* axes colored as red/green/blue arrows, respectively) resulting from CASSCF calculations. Semicoordinated oxygen atoms are depicted with dotted lines in the case of **5Ce**.

First, the DFT method with the ωB97X-V range-separated hybrid functional supplemented by quantum theory of atoms in molecules (QT-AIM)^89,90^ was employed to investigate the coordination bonds of these lanthanide complexes. With the help of the AIMAII software, the critical points of the type (3, −1) so-called bond critical points (BCPs) were identified by the analysis of electron density ρ­(**r**). Next, several functions were calculated at these BCPs, namely, the Laplacian of the electron density [∇^2^ ρ (**r**)], electron kinetic energy [*G*(**r**)], potential energy density [*V*(**r**)], and electron energy density [*h*_e_(**r**)]. Moreover, the coordination bonds were also characterized by calculating the AIM delocalization index (DI),^91^ which provides a quantitative measure of the sharing of electrons between atoms. Usually, the DI adopts a value close to 1 for a single covalent bond. First, QT-AIM was applied to 3Nd, and eight BCPs were found in the vicinity of the central Nd central atom. The quantitative results are summarized in Table 1, from which it can be concluded that there are eight coordination Nd–O bonds because it holds ∇^2^ *r*(**r**) > 0, *h*_e_(**r**) < 0, and also, it holds |*V*(**r**)|/*G*(**r**) > 1. Thus, the QT-AIM criteria for the definition of a coordination bond are fulfilled.^92^ By comparing the DI values, we can conclude that the nitrate ligands are much more weakly bonded to the central atom than the phenolate atoms of ligands **L1**.

**Table 1:** Results of QT-AIM Analysis for the Molecular Fragment of **3Nd**

bond	DI	ρ (*r*)	∇^2^ ρ (*r*)	*h*_e_ (*r*)	|*V*(*r*)|/*G*(*r*)
Nd1–O2	0.284	0.0580	0.2424	–0.00304	1.048
Nd1–O2	0.284	0.0584	0.2426	–0.00319	1.050
Nd1–O2	0.285	0.0581	0.2424	–0.00300	1.047
Nd1–O2	0.284	0.0580	0.2414	–0.00303	1.048
Nd1–O4	0.176	0.0368	0.1356	–0.00032	1.009
Nd1–O4	0.176	0.0366	0.1360	–0.00020	1.006
Nd1–O4	0.177	0.0367	0.1359	–0.00019	1.005
Nd1–O4	0.176	0.0368	0.1358	–0.00033	1.009

Analogous analysis was also performed for **5Ce**, and the results are listed in Table 2. For this complex, the DI of the phenolate atoms of ligand **L2** has similar values to those found for **3Nd**. However, DI values for nitrate ligands suggest that weaker bonds were formed. Based on QT-AIM analysis, it can be concluded that the phenolate atoms of ligand **L2** form coordination bonds to the Ce atom [∇^2^ *r*(**r**) > 0, *h*_e_(**r**) < 0, and |*V*(**r**)|/*G*(**r**) > 1]. However, this is not true for the oxygen atoms of nitrate ligands. Herein, ∇^2^ *r*(**r**) > 0, *h*_e_(**r**) > 0 and, concurrently, |*V*(**r**)|/*G*(**r**) < 1, which means that these oxygen atoms are semicoordinated to the Ce­(III) ion. From this analysis, we can presume that the dominant part of the ligand field is created by the phenolate atoms of **L2** ligands. This is in accordance with the orientation of the g_*z*_ vector oriented midway between phenolate atoms and the oblate nature of the electronic ground state of the Ce^III^ ion (Figure 7), and such a strong axial ligand field is also responsible for the large energy splitting observed in Figure S20. Although the Ce–O_nitrato_ bond distance (2.684 Å, Table S2) is just slightly above average for all of the reported compounds featuring Ce–nitrato coordination bonds (Figure S21), the present work represents the first case of a Ce–O semicoordination bond being elucidated through a QT-AIM analysis. However, whether the compounds with longer Ce–O distances present such semicoordination remains unknown.

**Table 2:** Results of QT-AIM Analysis for the Molecular Fragment of **5Ce** ^a^

bond	DI	ρ (*r*)	∇^2^ ρ (*r*)	*h*_e_ (*r*)	|*V*(*r*)|/*G*(*r*)
Ce1–O1	0.281	0.0539	0.2029	–0.00258	1.048
Ce1–O1	0.281	0.0538	0.2025	–0.00255	1.048
Ce1–O1	0.281	0.0538	0.2044	–0.00257	1.048
Ce1–O1	0.281	0.0538	0.2045	–0.00256	1.048
Ce1–O3	0.158	0.0318	0.1138	0.00064	**0.977**
Ce1–O3	0.158	0.0318	0.1139	0.00065	**0.977**
Ce1–O3	0.158	0.0319	0.1141	0.00061	**0.978**
Ce1–O3	0.158	0.0319	0.1140	0.00061	**0.978**

a The |*V*(*r*)|/*G*(*r*) values in bold indicate the presence of semicoordination between the Ce­(III) ion and the oxygen from the nitrato ligands.

Next, the CASSCF calculations were done for the complexes **3Nd** and **5Ce** and also for the molecular fragment in **3Nd**, in which the Nd atom was replaced by a Ce atom (herein labeled as **3Ce**′) as the SC-XRD analysis was not possible for the **3Ce** compound. Besides classical CASSCF calculations (herein labeled as method A), CASSCF/NEVPT2 was applied to Ce­(III) complexes (method B). The details of these calculations are described in the Experimental Section. The respective energies of the ligand field multiplets originating from ^4^I_9/2_ for Nd­(III) and ^2^F_5/2_ for Ce­(III) ions are shown in Figure S20. It is obvious that in the case of **3Nd**, the lowest Kramers doublet (KD) is located at ≈60 cm^–1^, whereas a much larger energy separation, ca. 600 cm^–1^, is found for Ce­(III) ions in **3Ce**′ and **5Ce** (Figure 8). Such an energy separation is considerably larger than that for the majority of Ce^III^ compounds reported to date; a detailed discussion and comparison of Ce^III^ compounds can be found in ref 35. The orientation of the g-tensor axis of the ground-state KD is shown in Figure 7. Moreover, the SINGLE_ANISO module was utilized to calculate ab initio magnetization blocking barriers for these complexes, and the results are summarized in Figure S20 and Table 3. The computed probabilities for the QTM of the ground state are high enough for the Ce^III^ so that a small static field is needed to observe a slow relaxation of the magnetization, which is in accordance with the AC susceptibility experiments. Moreover, the temperature-dependent DC magnetic data are depicted in Figure S22, which are in good agreement with the experimental measurements.

**Figure 8:**
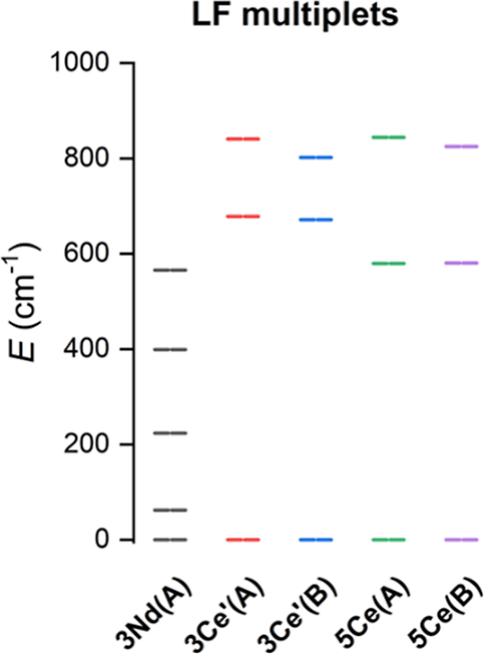
Output of the CASSCF calculations for molecular fragments of **3Nd**, **3Ce**′, and **5Ce**. The plot shows LFM stemming from the ^4^I_9/2_ state for Nd­(III) and ^5^F_5/2_ state for Ce­(III) ions.

**Table 3:** Results of CASSCF (Method A) and CASSCF/NEVPT2 (Method B) Calculations for **3Nd**, **3Ce**′, and **5Ce**

	3Nd(A)	3Ce′(A)	3Ce′(B)	5Ce(A)	5Ce(B)
*g*-tensor of 1st KD	0.046	0.015	0.030	0.143	0.177
	0.071	0.648	0.716	0.756	0.846
	5.163	3.612	3.577	3.681	3.633
tunneling rate of 1st KD	0.019	0.111	0.124	0.150	0.170
Δ*E*_1–2_ (cm^–1^)	62	678	671	580	580

Finally, it is noteworthy that despite Δ*E*_1–2_ being higher for **3Ce**′ than for **5Ce** (Table 3), the calculated energy barrier is slightly higher for **4Ce** than for **1Ce** (see above), elucidating the fact that such an energy barrier difference might have its origin not in the electronic states but in the difference between the local mode energies (12.60 and 13.46 cm^–1^ for **1** and **4**, respectively). We hypothesize that such a difference in the local mode energy might arise from the slight difference in rigidities between the ethylene and cyclohexane moieties in ligands **H**_**2**_ **L1** and **H**_**2**_ **L2**, respectively.

## Conclusions

In this work, five new Ce^III^-based MOFs were synthesized and magnetically and spectroscopically characterized. These frameworks, containing different encapsulated counterions, can be built with an easy one-pot synthesis employing different Schiff base ligands, which are coordinated in a bridge-like unusual way. The counteranions lie in the center of the pores, and the frameworks are isostructural across counteranions. These frameworks show field-induced slow relaxation of magnetization, which is relatively unusual for Ce^III^-based systems. Although the relaxation dynamics of these frameworks could be fit to many different combinations, the study of this relaxation at different applied magnetic fields allowed us to find a combination of local modes and direct processes, with a contribution of QTM at low fields. By using a magnetically diluted Ce^III^ in a diamagnetic La^III^ matrix, the source of QTM was found to be spin–spin dipolar interactions, leading to the observation of a maximum at fields as low as 0.002 T, without any visible contribution of QTM. At intermediate fields, a Raman-type relaxation dominates, and the relaxation time starts plummeting once the direct pathway starts to dominate at high fields. Deeper analysis of the temperature dependence of the *d* parameter reveals that the Raman type relaxation takes place through a well-defined vibrational mode at 12.84 cm^–1^, which is in perfect agreement with the local mode energy value found at 12.64 cm^–1^ and with the vibrational mode experimentally found through ultralow-frequency Raman spectroscopy at 12.28 cm^–1^.

QT-AIM calculations elucidated the presence of a semicoordination bond between the nitrato ligands and the Ce^III^ ions, which, along with the strong phenolato donors, creates an optimal ligand field with a very large energy separation between the ground and first excited KDs, with the *g*_*z*_ tensor oriented along such phenolato atoms. This semicoordination was found not to be present in the Nd^III^ analogue.

In conclusion, this work provides one of the first experimental evidence of a local mode effectively generating a vibronic barrier and sheds some light on the missteps taken when applying the optical–acoustic mechanism to anomalous Raman exponents. Further unveiling the relaxation dynamics of Ce^III^-based compounds paves the way for their possible application as quantum bits in quantum information processing, where a deep understanding of the spin–phonon relaxation dynamics is needed to fully control and lengthen their decoherence times.

## Supplementary Material



## Notes

Additional synthetic, structural, magnetic, and spectroscopic data (PDF)
