# Supplementary material for: Cerium-Based Metal–Organic Frameworks: Unveiling the Role of Terahertz Vibrations in the Spin Relaxation Dynamics
Source: Inorg Chem. 2025 Feb 18;64(8):3735–46. doi: 10.1021/acs.inorgchem.4c04542 (PMC12164259; doi:10.1021/acs.inorgchem.4c04542)
Supplement: Supplementary file 1 [file ic4c04542_si_001.pdf]

Supporting Information

"Cerium-based Metal-Organic Frameworks with Helical Chirality: Unveiling the Role of Terahertz Vibrations in the Spin Relaxation Dynamics"

Joan Torrent, [§] **Cristina Puigjaner,**[¥] **Radovan Herchel**[γ]**, Júlia Mayans**[*§]

§ Departament de Química Inorgànica i Orgànica, Secció Inorgànica and Instutut de Nanociència i Nanotecnologia (IN2UB). Universitat de Barcelona, Martí i Franquès 1-11, Barcelona-08028, Spain.

γ Department of Inorganic Chemistry, Faculty of Science, Palacký University, 77147 Olomouc, Czech Republic

¥ Departament de Mineralogia, Cristal·lografia i Dipòsits Minerals, Universitat de Barcelona, Martí Franqués s/n, 08028 Barcelona (Spain) and Unitat de Difracció de R-X. Centre Científic i Tecnològic de la Universitat de Barcelona (CCiTUB), Solé i Sabarís 1-3. 08028 Barcelona.

Corresponding Author E-mail: julia.mayans@ub.edu

Table of contents

1- Structural information

Table S1: Crystallographic data for the X-ray structure of **3Nd**.

Figures S1-S3: Packing of **3Nd** along the a, b, and c crystallographic axes.

Table S2: Selected bond lengths and angles for compounds **3Nd** and **5**.

Figure S4: IR spectra of compounds **1-6**.

Figure S5: PXRD spectra.

Figure S6: Coordination polihedra around the Ln^{III} cation.

2- DC data

Figure S7: Reduced magnetization plots for compound **1**.

3- AC data

Figure S8: Temperature dependence of the imaginary component of compounds **1**, **2Nd** and **4** at different applied fields at a fixed frequency of 1000 Hz.

Figure S9: Temperature dependence of the imaginary component of compound **1Ce20%@La** at different applied fields and at a fixed frequency of 1000 Hz.

Figure S10: Temperature dependence of the imaginary component of compounds **1** and **4** at different frequencies at an applied field of 0.02 T.

Figure S11: Evolution of the alpha parameter for compounds **1** and **4** at different temperatures at a constant field of 0.02 T.

Figure S12: log-log plot for compounds **1** and **4** at a field of 0.02 T.

Figure S13: Frequency dependence of the in-phase and out-of-phase magnetic susceptibility components of **1** at different temperatures and fixed DC fields.

Figure S14: Cole-Cole plots of **1** at different temperatures and different applied DC fields.

Figure S15: ln(τ) vs the inverse of temperature at different DC fields for compound **1**.

Figure S16: Frequency dependence of the in-phase and out-of-phase components of the magnetic susceptibility of compound **1** at a fixed temperature and different DC fields.

Figure S17: Cole-Cole plots of compound **1** at a fixed temperature and different DC fields.

Figure S18: Inverse of τ dependence on the magnetic field extracted from ac susceptibility measurements at different temperatures.

Figure S19: Arrhenius plot of the *d* parameters extracted from the Brons-van Vleck model.

Table S3: Different combinations of relaxation pathways employed to fit the ac data from compounds **1** and **4** at 0.02 T.

Table S4: τ vs T values for compound **1** at the various applied DC fields.

Table S5: Best fitting parameters to describe the temperature dependence of the relaxation rate for compound **1**.

Table S6: Best fits to reproduce the field dependence of the relaxation rate for compound **1.**

4- Theoretical calculations

Figure S20: Magnetization blocking barrier for compounds **3Nd**, **3Ce’** and **5Ce**.

Figure S21: Ce-$O_{nitrato}$ bond distance histogram of the reported CCDC structures featuring Ce-Nitrato coordination bonds.

Figure S22: Theoretical magnetic susceptibilities for compounds **3Nd**, **3Ce’**, and **5Ce**.

1- Structural information

Table S1: Crystallographic data of the X-ray structure of **3Nd.**

	**1Nd**
**Formula**	$C_{36}H_{40}BF_4N_6NdO_{16}$
**FW**	1043.79
**System**	Hexagonal
**Space group**	P 62 2 2
**a/Å**	16.2710(12)
**b/Å**	16.2710(12)
**c/Å**	16.5288(14)
**α/deg.**	90
**β/deg.**	90
**γ/deg.**	120
**V/Å3**	3789.7(6)
**Z**	3
**T, K**	100(2)
**λ(MoKα), Å**	0.71073
**ρ_{calc}, g•cm^{-3}**	1.372
**μ(MoKα), mm^{-1}**	0.959
**F (000)**	1581
**R**	0.0669
**ωR^2**	0.1593

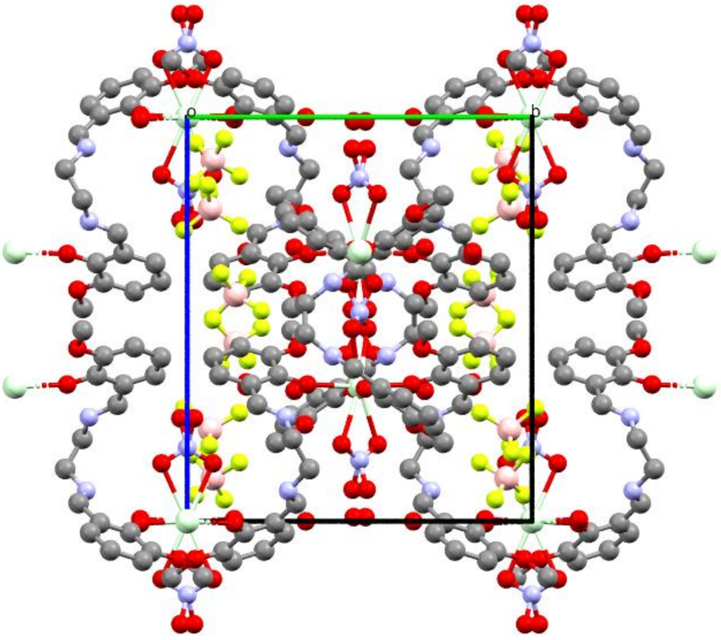

Figure S1: Crytal packing of **3Nd** along the *a* crystallographic axis. Color code: Blue: Nitrogen, Gray: Carbon, Green: Fluoride, Pink: Boron, Turquoise: Neodymium, Red: Oxygen. Hydrogen atoms have been omitted for clarity.

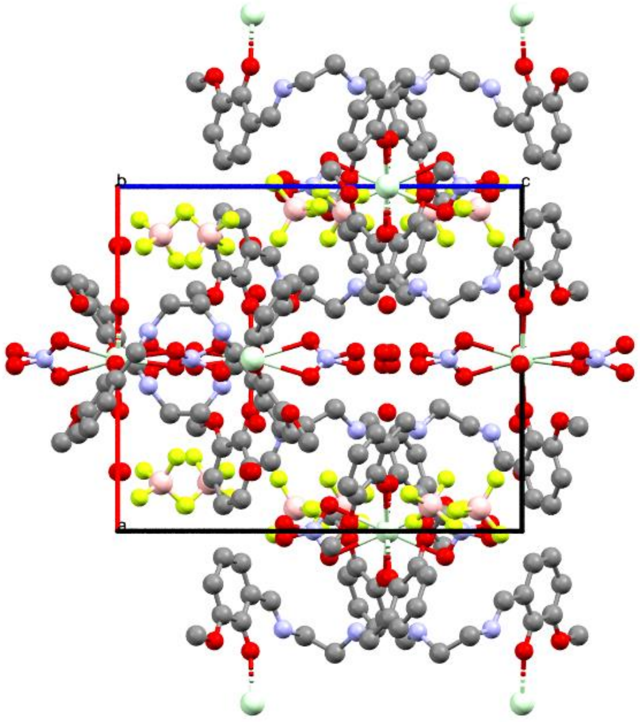

Figure S2: Crystal packing of **1Nd** along the *b* crystallographic axis. Color code: Blue: Nitrogen, Gray: Carbon, Green: Fluoride, Pink: Boron, Turquoise: Neodymium, Red. Oxygen. Hydrogen atoms have been omitted for clarity.

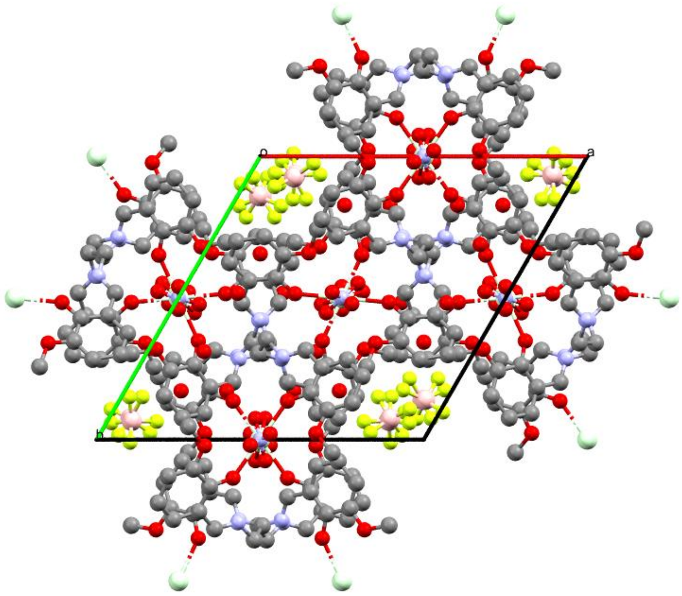

Figure S3: Crystal packing of **1Nd** along the *c* crystallographic axis. Color code: Blue: Nitrogen, Gray: Carbon, Green: Fluoride, Pink: Boron, Turquoise: Neodymium, Red. Oxygen. Hydrogen atoms have been omitted for clarity.

	3Nd	5Ce
Ln-O3	2.593	2.684
Ln-O1	2.352	2.413
O1-Ln-O1$_{(trans)}$	168.98	174.37
O1-Ln-O1$_{(cis)}$	48.84	46.90
O1-Ln-O3	114.55	111.05
O3-Ln-O1	69.53	77.21
O3-Ln-O3$_{(trans)}$	175.87	171.39
O3-Ln-O3$_{(cis)}$	71.32	77.12
Ln-Ln-Ln	111.79	101.90
N-C-C-N	69.24	67.10

Table S2: Selected bond distances (Å), angles (º), and torsions (º) for compounds **3Nd** and **5**, the latter first reported in Ref. 1.

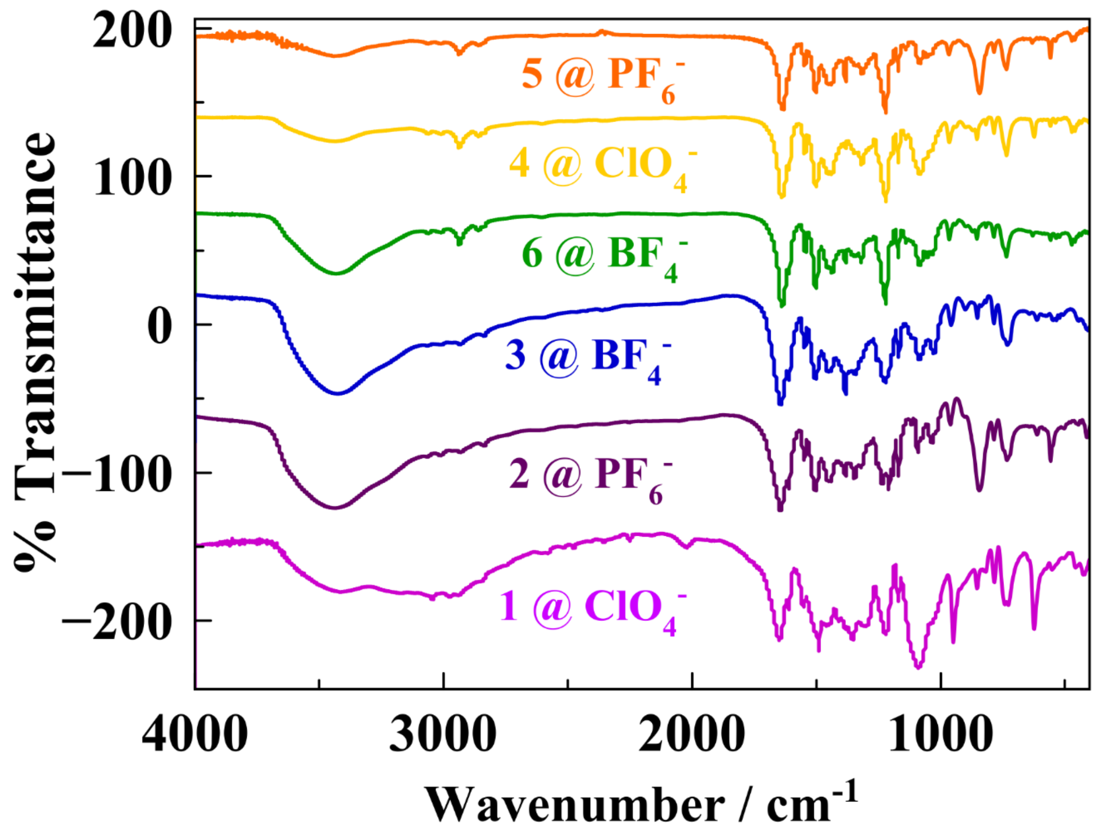

Figure S4: Infrared spectra of compounds **1-6**.

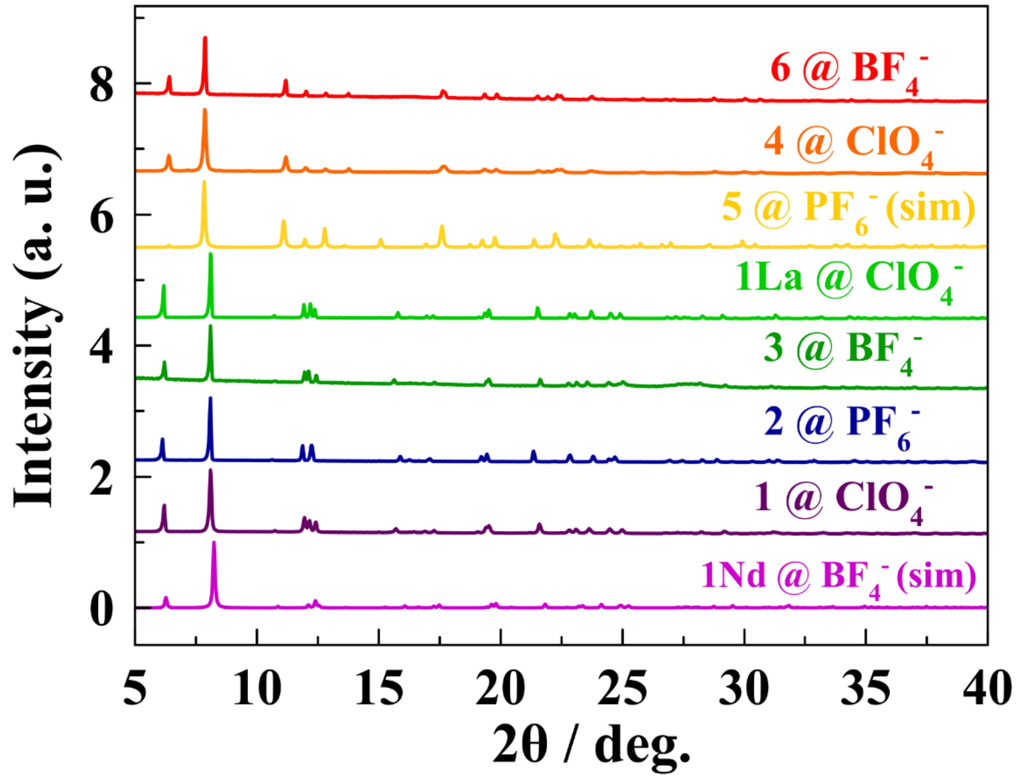

Figure S5: PXRD patterns of compounds **1-6**, as well as of the diamagnetic analogue **1La**.

Figure S6: Coordination polihedra around the Ln^{III} cation for **3Nd** (left) and **5** (right).

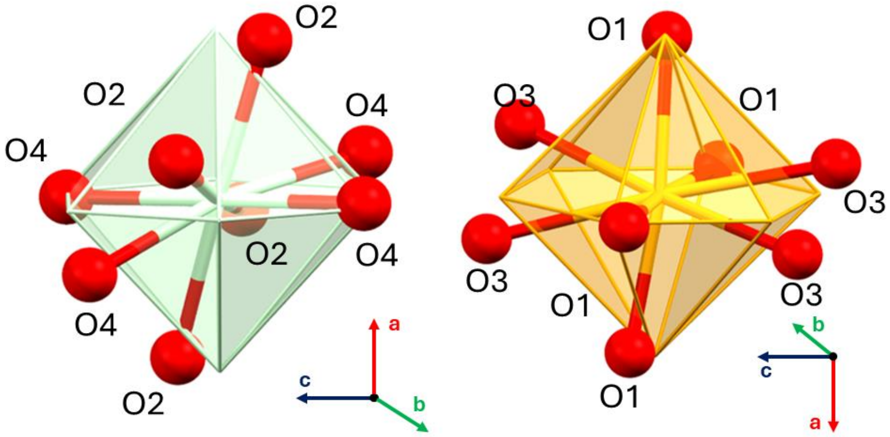

2- DC data

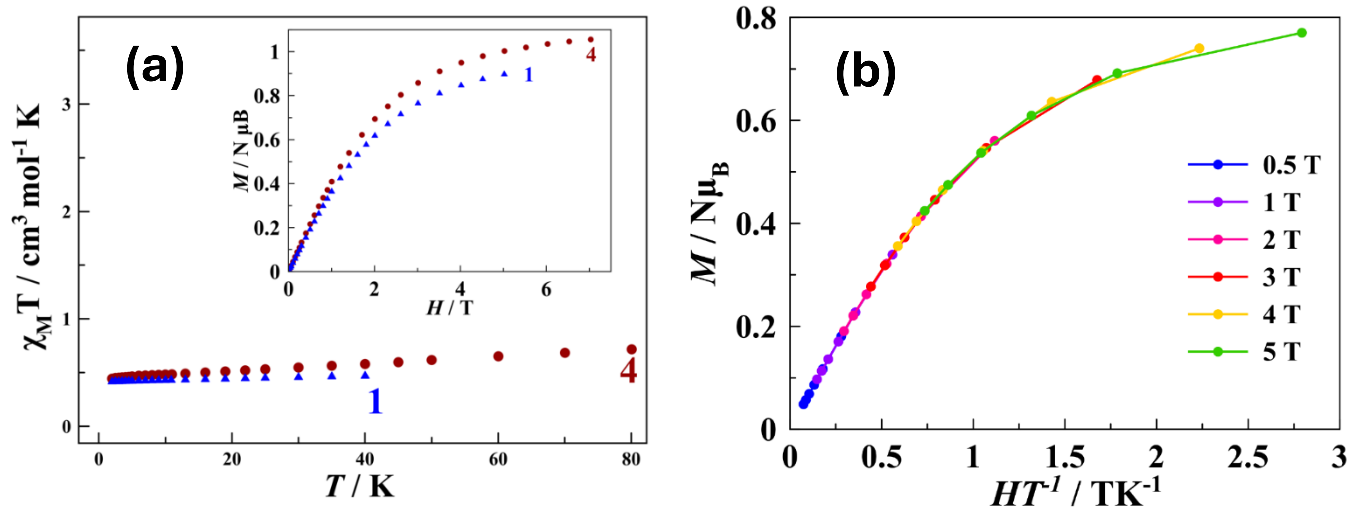

Figure S7: (a) $\chi_M T$ *vs* *T* product for compounds **1** and **4**, the inset shows the magnetization curves for both compounds. (b) Reduced magnetization plot of compound **1.**

3- AC data

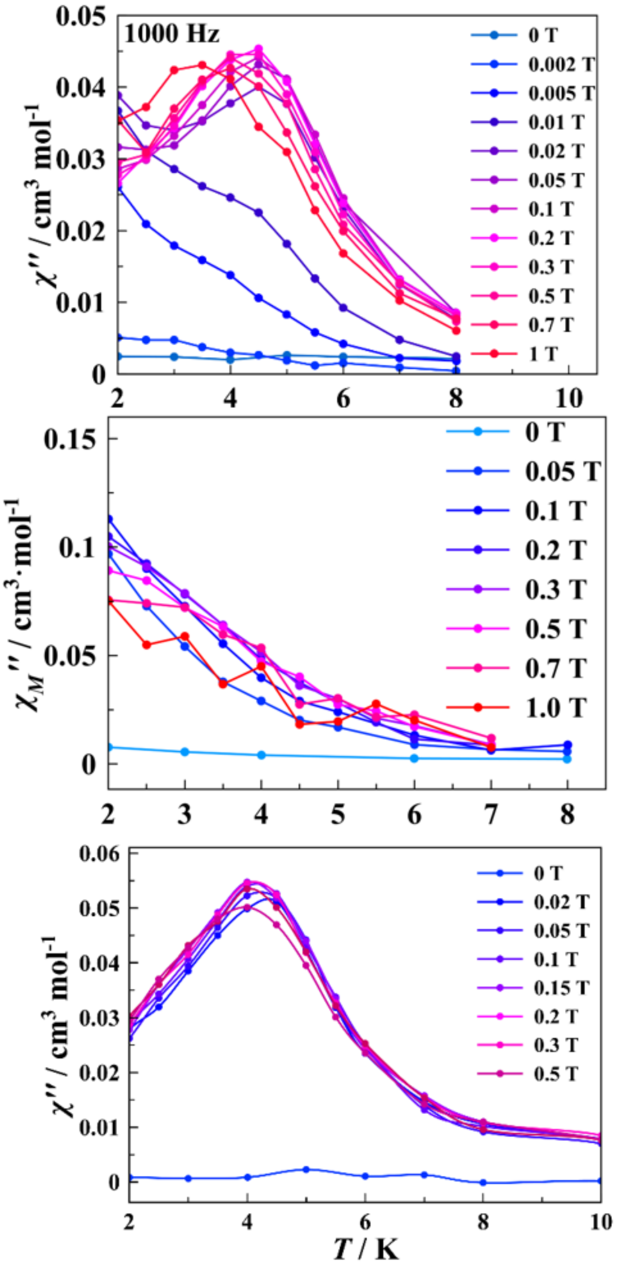

Figure S8: Temperature dependence of the imaginary component of the magnetic susceptibility for compounds **1** (top)**, 2Nd** (middle) and **4** (bottom) at different applied fields and a fixed frequency of 1000 Hz.

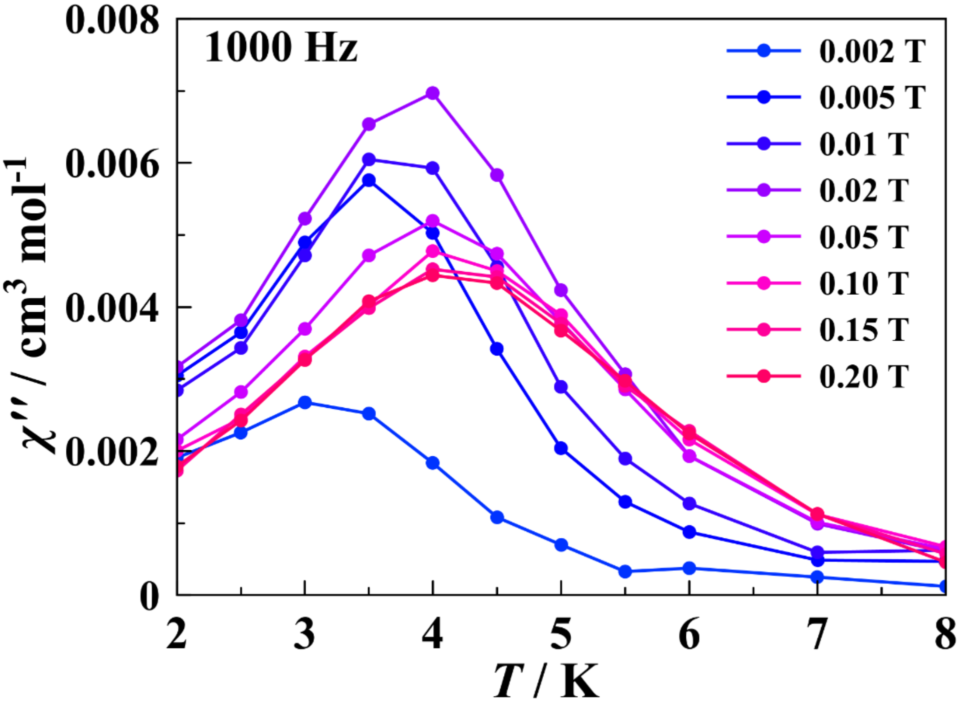

Figure S9: Temperature dependence of the imaginary component of the magnetic susceptibility for compound **1Ce20%@La** at different applied fields at a fixed frequency of 1000 Hz.

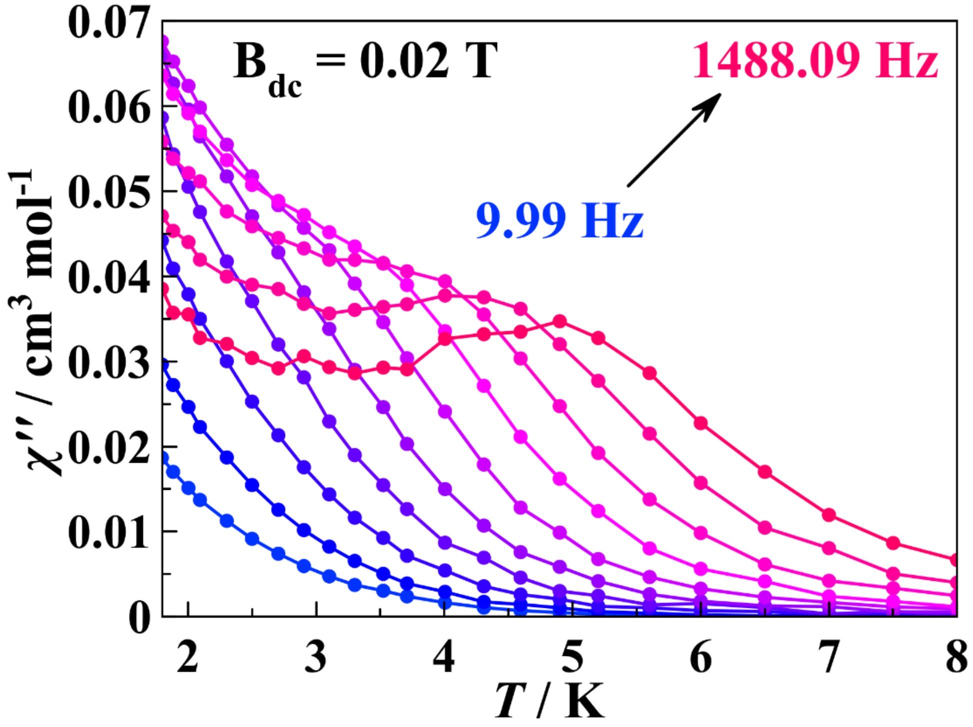

Figure S10: Temperature dependence of the imaginary component of the magnetic susceptibility at a fixed field at 0.02 T and variable frequency.

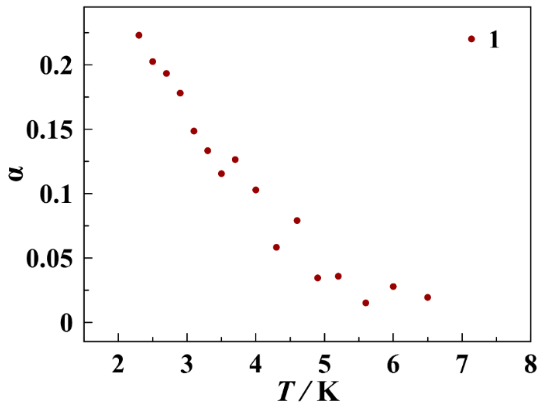

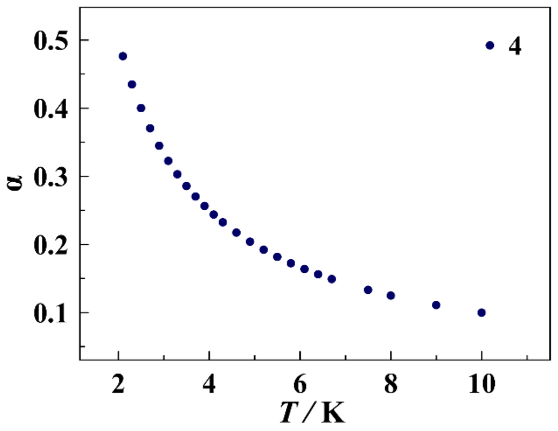

Figure S11: Evolution of the alpha parameter for compound **1** at an applied field of 0.02 T.

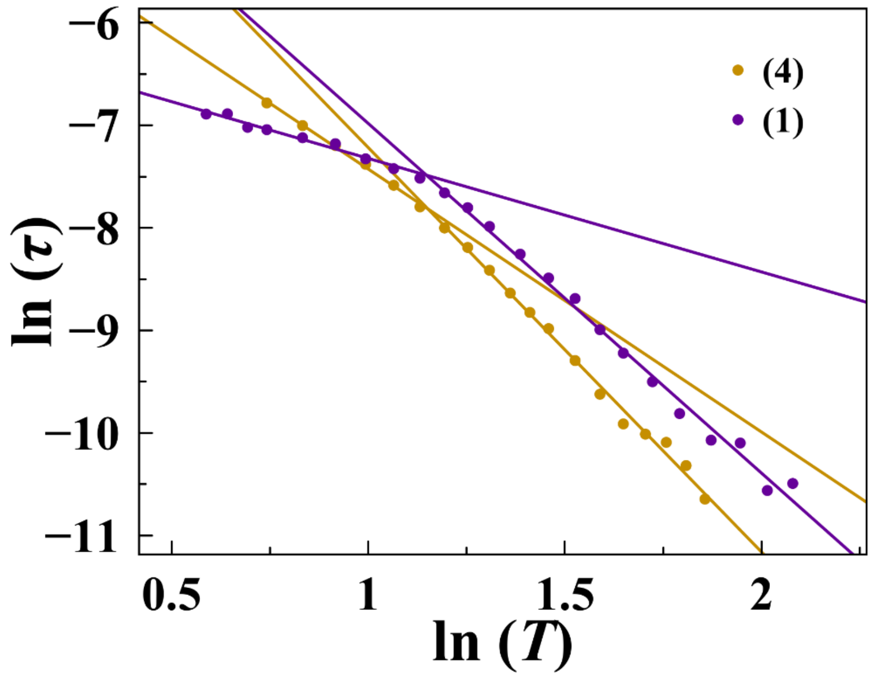

Figure S12: log-log plot for compounds **1** and **2** at a field of 0.02 T.

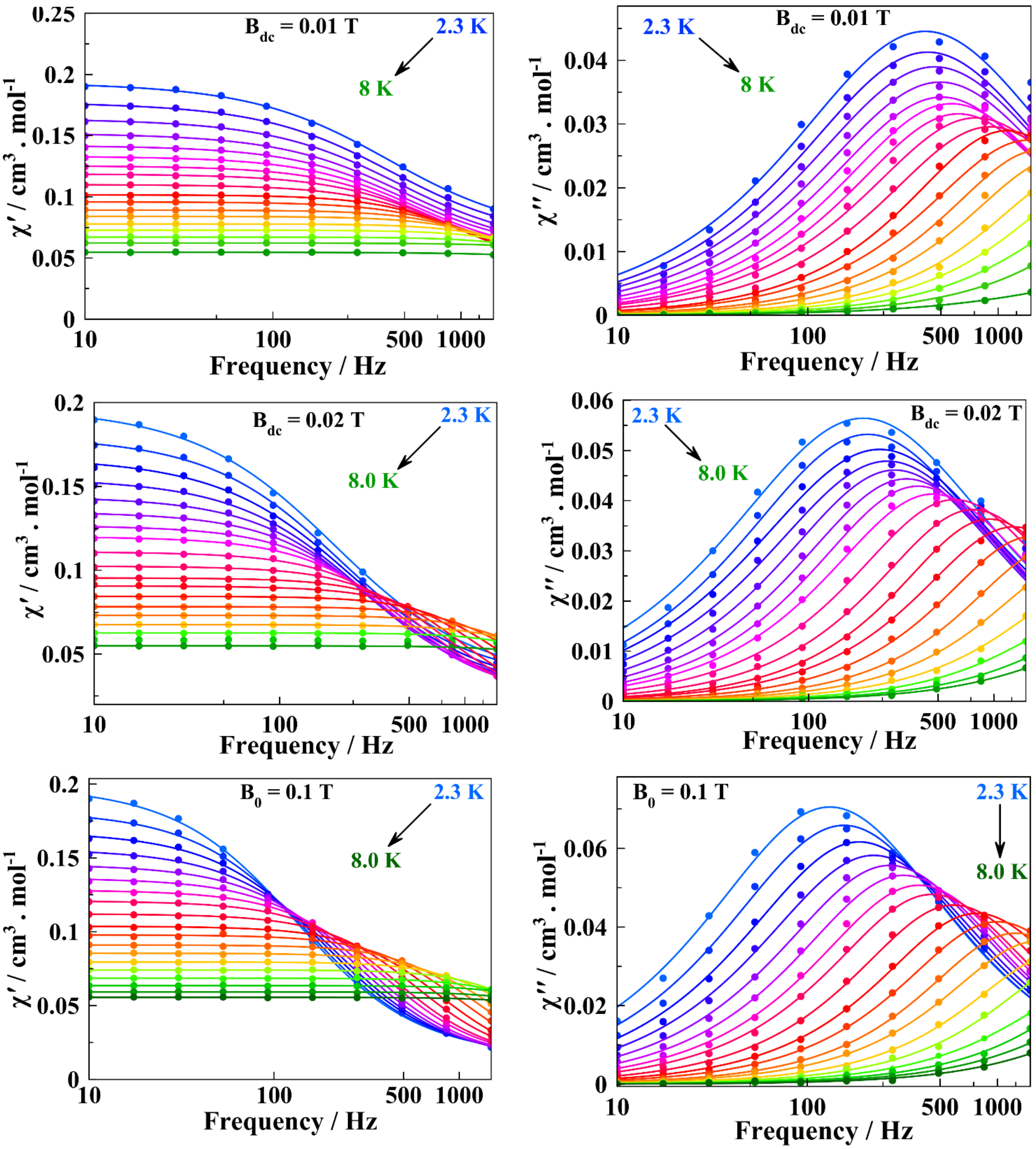
B_dc = 0.01 T
2.3 K
8 K
χ' / cm3 . mol-1
χ'' / cm3 . mol-1
Frequency / Hz
B_dc = 0.02 T
8.0 K
B_0 = 0.1 T

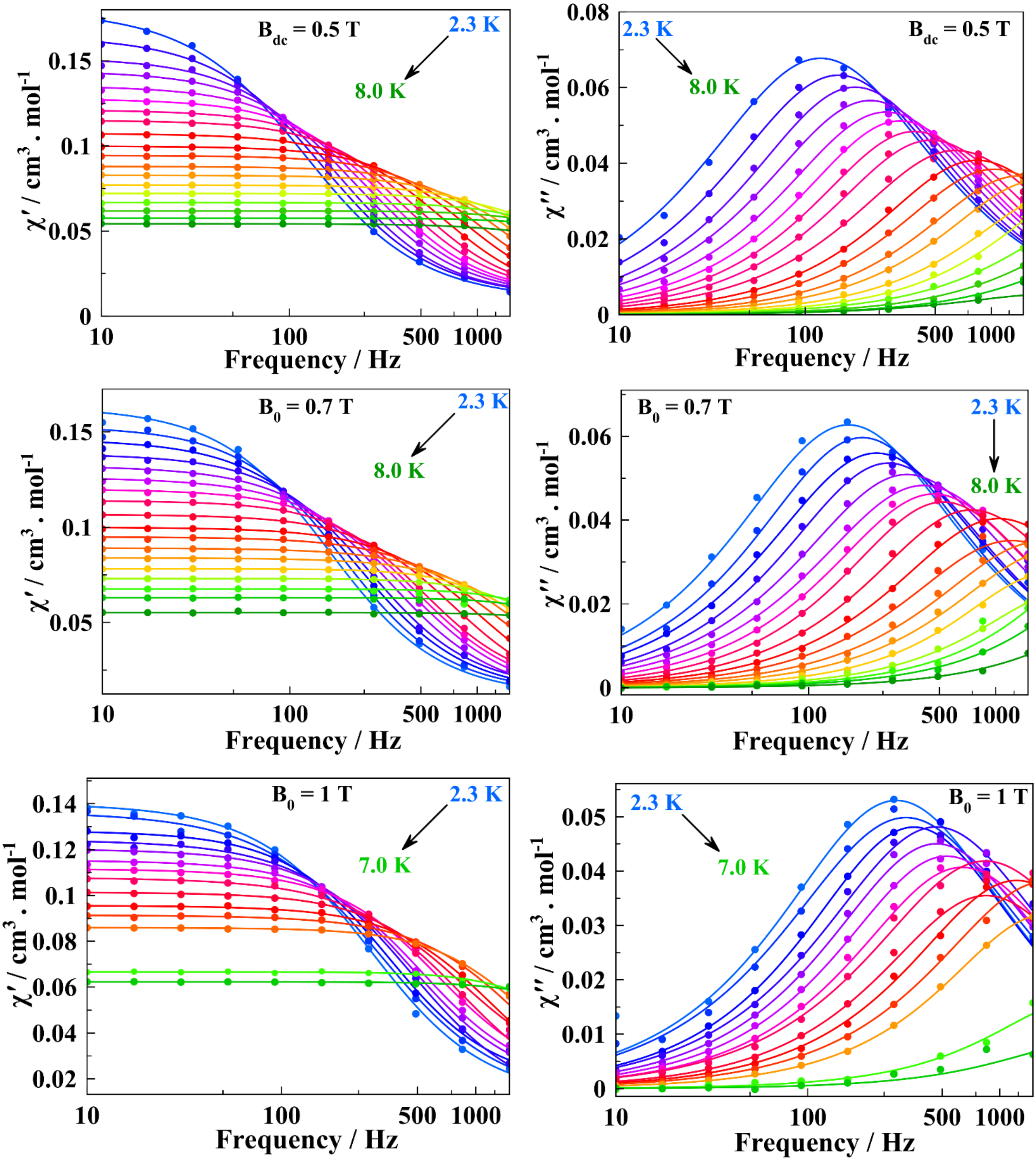

B_{dc} = 0.5 T
2.3 K
8.0 K
0.15
0.1
0.05
0
χ' / cm³ . mol⁻¹
10
100
500
1000
Frequency / Hz
0.08
0.06
0.04
0.02
χ'' / cm³ . mol⁻¹
B_0 = 0.7 T
0.12
0.14
B_0 = 1 T
7.0 K
0.03
0.01

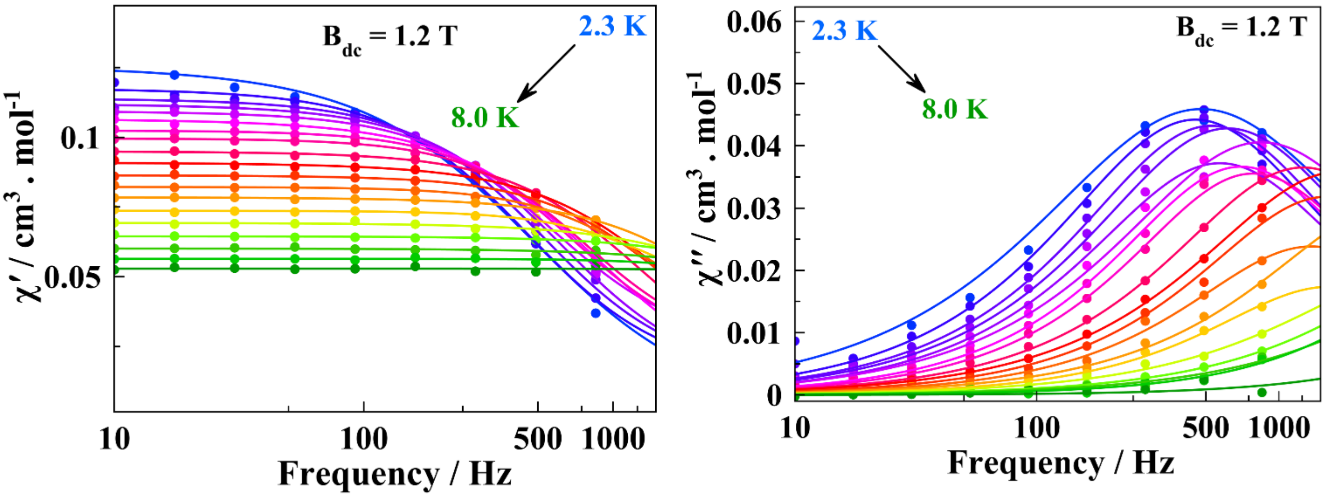

Figure S13: Frequency dependence of the in-phase (left) and out-of-phase (right) components of the magnetic susceptibility at different temperatures and different fixed DC fields.

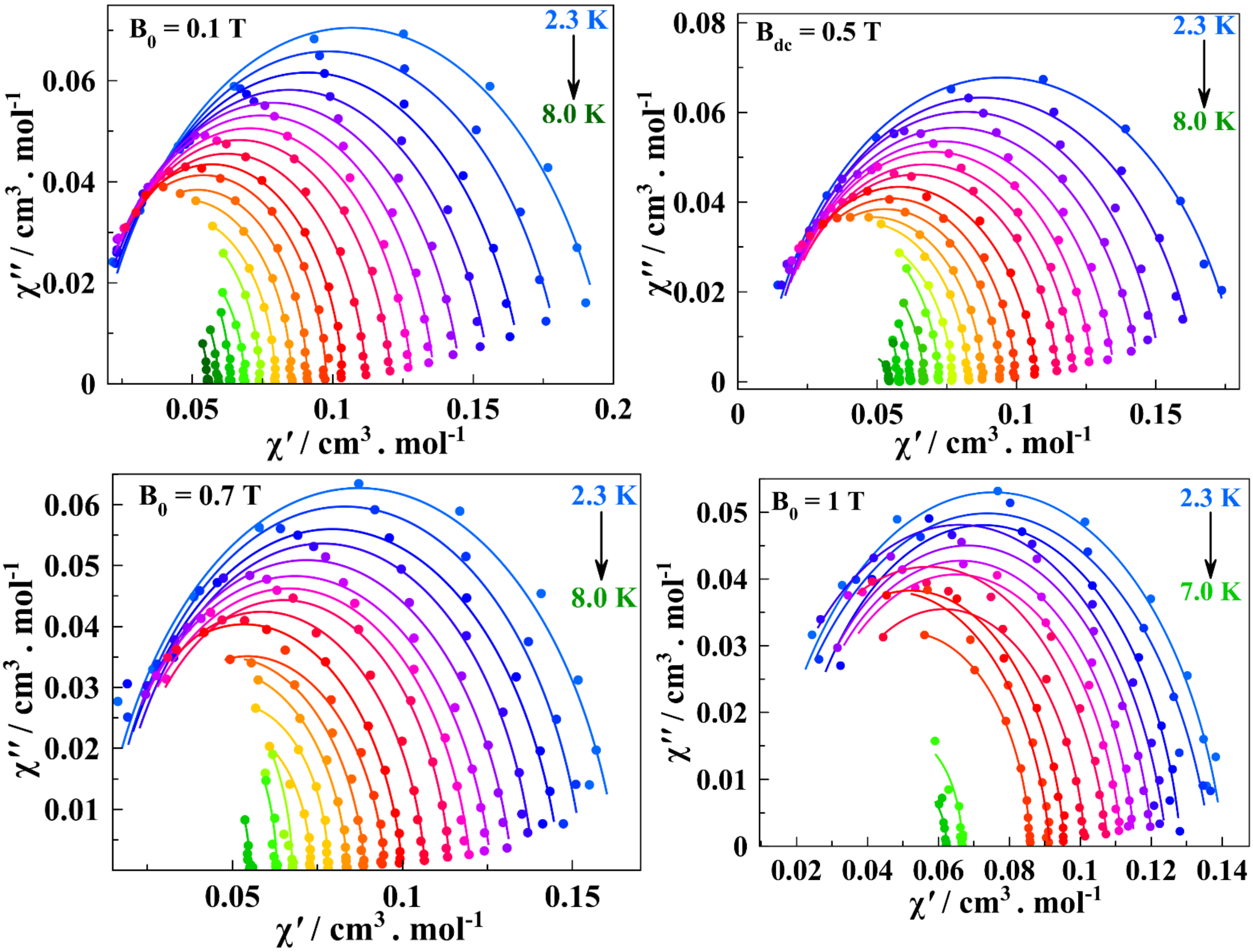

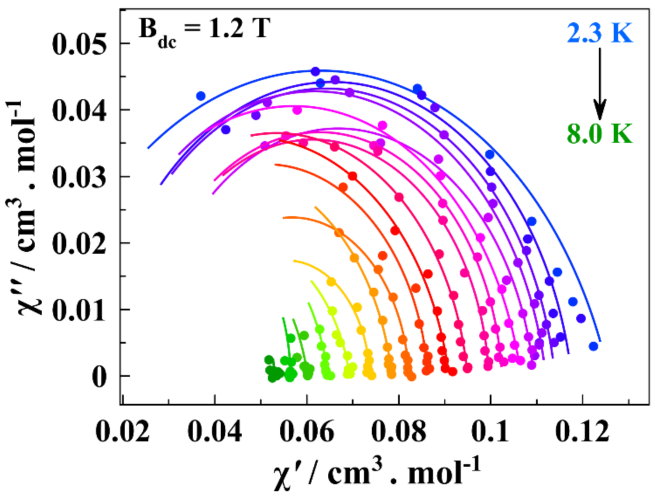

Figure S14: Cole-cole plots of compound **1** in the 2.3-8 K temperature range measured at different applied static fields.

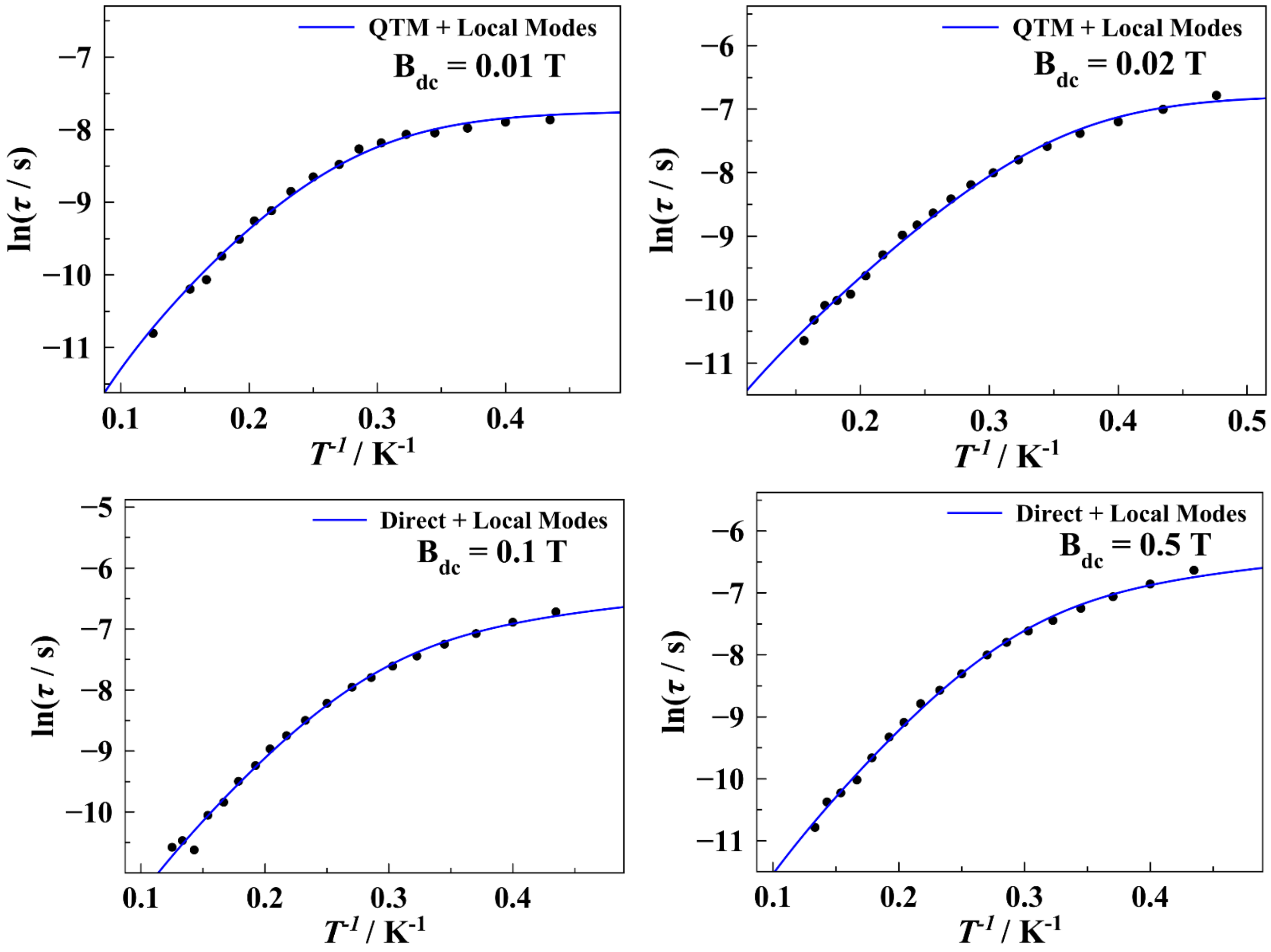

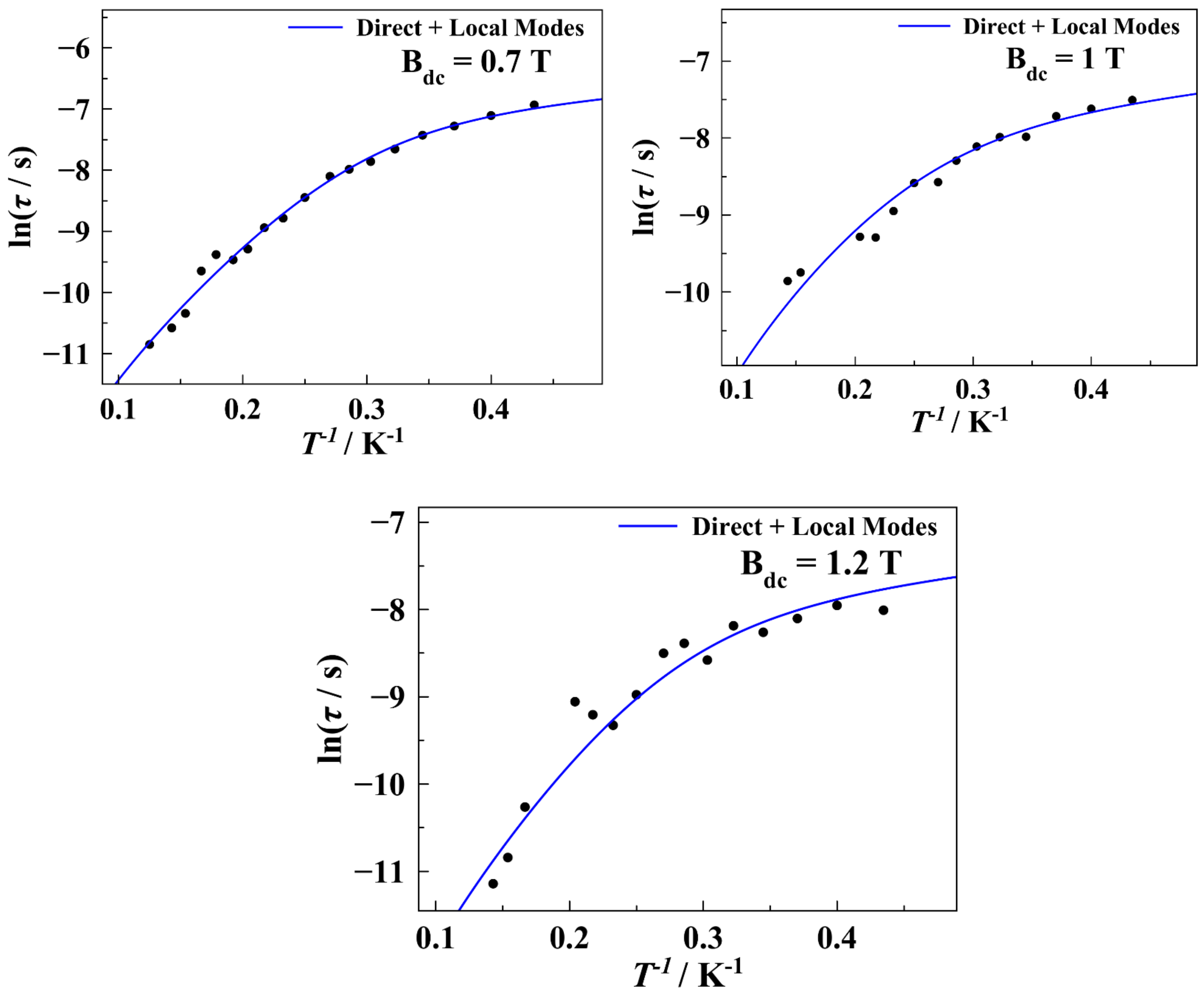

Figure S15: ln(τ) vs the inverse of the temperature in the different studied applied fields. Solid lines correspond to the best fits.

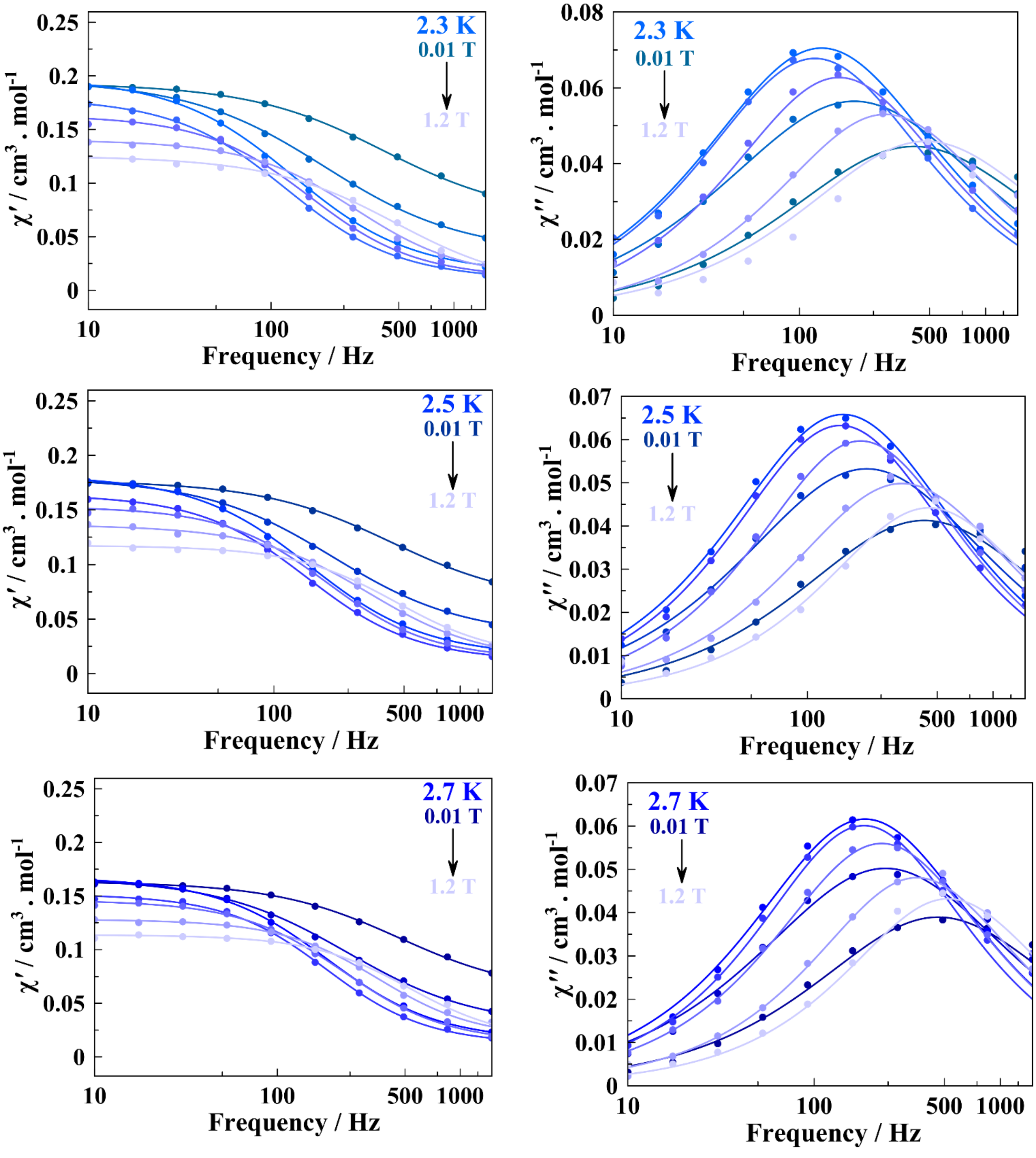
2.3 K
0.01 T
1.2 T
χ′ / cm3 . mol-1
χ′′ / cm3 . mol-1
Frequency / Hz
2.5 K
0.01 T
1.2 T
2.7 K
0.01 T
1.2 T
0.25
0.2
0.15
0.1
0.05
0
0.08
0.07
0.06
0.05
0.04
0.03
0.02
0.01
10
100
500
1000

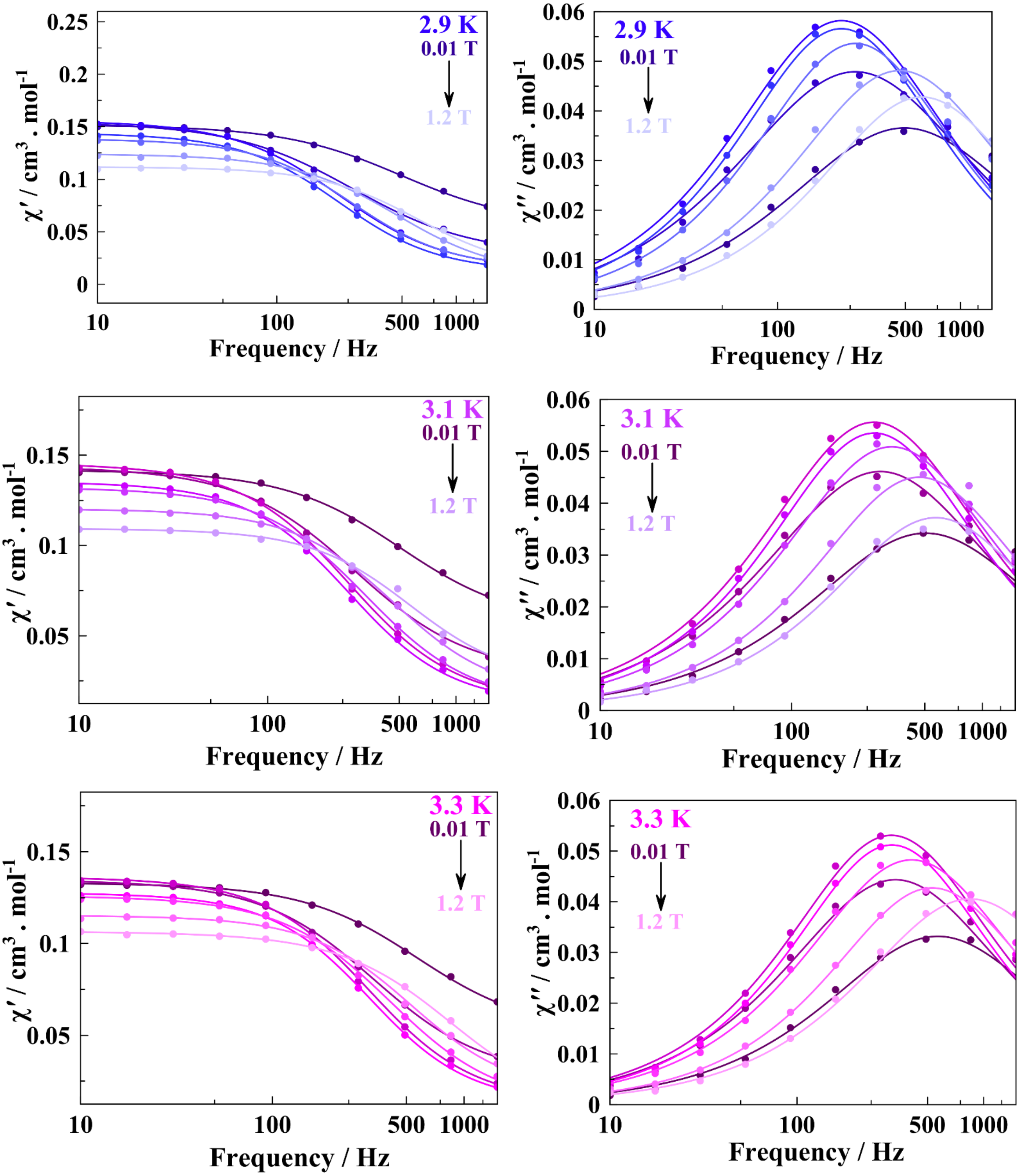
2.9 K
0.01 T
1.2 T
χ' / cm^3 . mol^{-1}
χ'' / cm^3 . mol^{-1}
Frequency / Hz
3.1 K
0.01 T
1.2 T
χ' / cm^3 . mol^{-1}
χ'' / cm^3 . mol^{-1}
Frequency / Hz
3.3 K
0.01 T
1.2 T
χ' / cm^3 . mol^{-1}
χ'' / cm^3 . mol^{-1}
Frequency / Hz

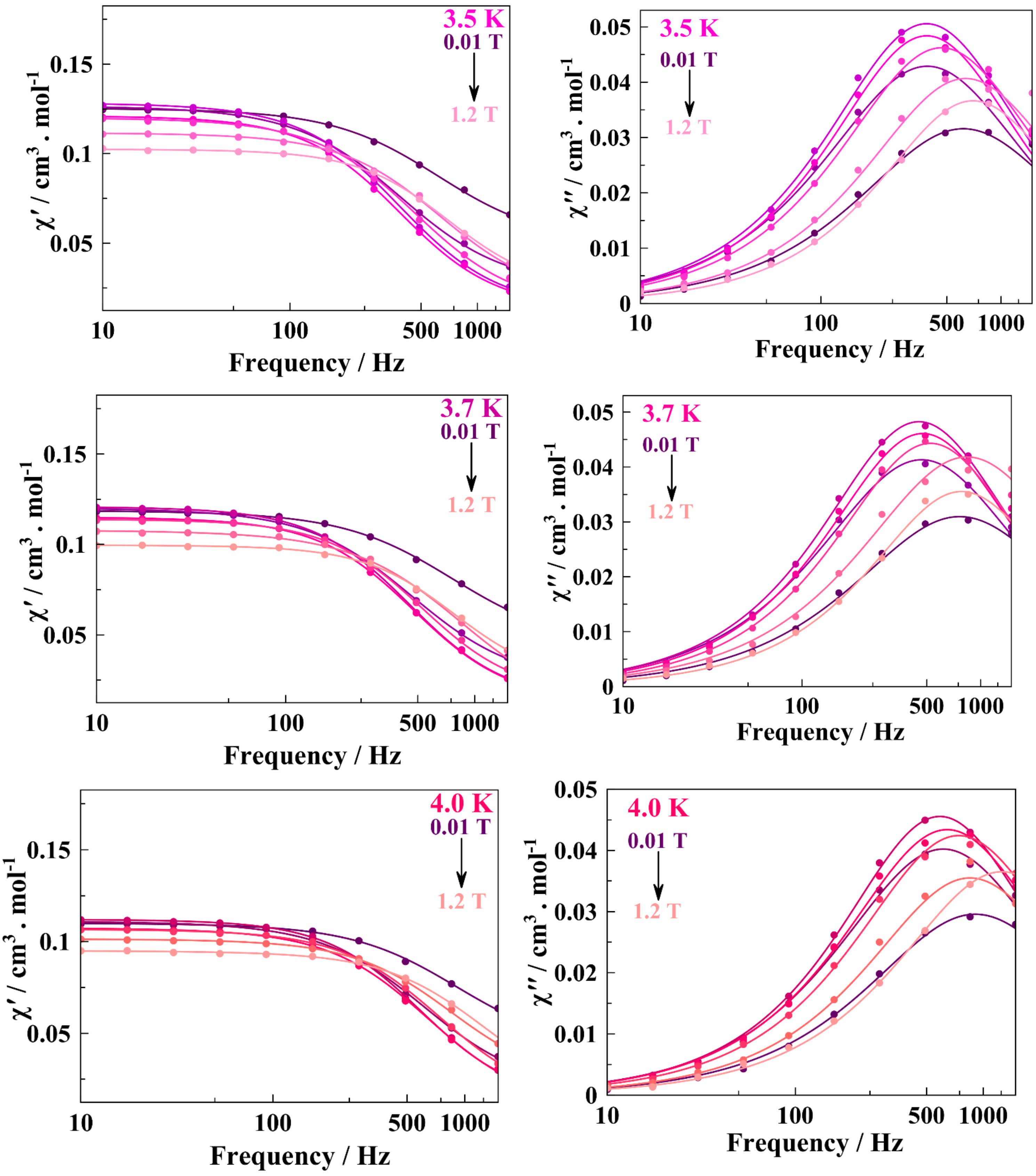
3.5 K
0.01 T
1.2 T
χ' / cm3 . mol-1
χ'' / cm3 . mol-1
Frequency / Hz
3.7 K
0.01 T
1.2 T
4.0 K
0.01 T
1.2 T

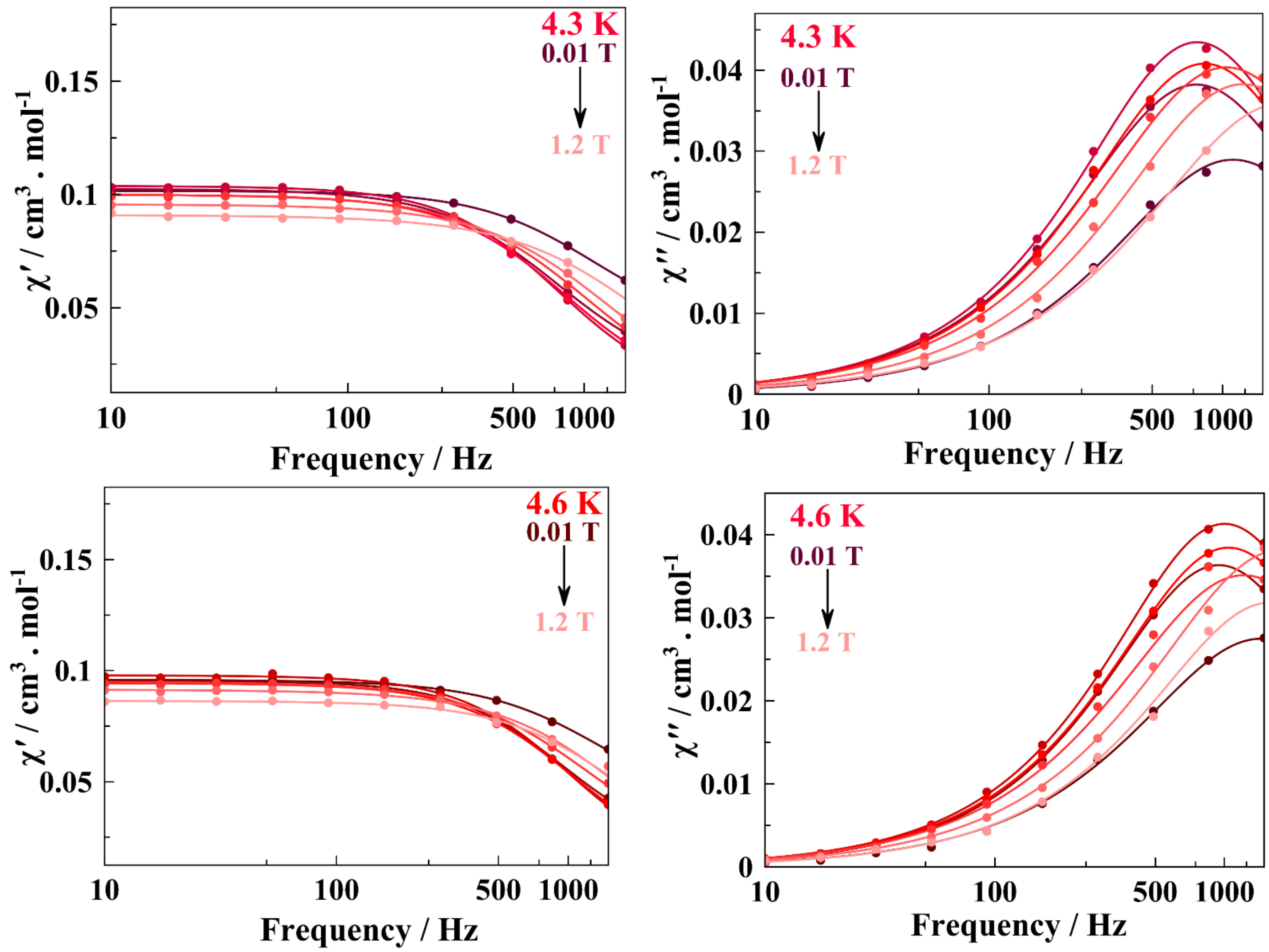

Figure S16: In-phase (left) and out-of-phase (right) components of the magnetic susceptibility recorded at a fixed temperature in the 0.01-1.2 T field range in the 10-1488 Hz frequency range. Solid lines indicate the best fits.

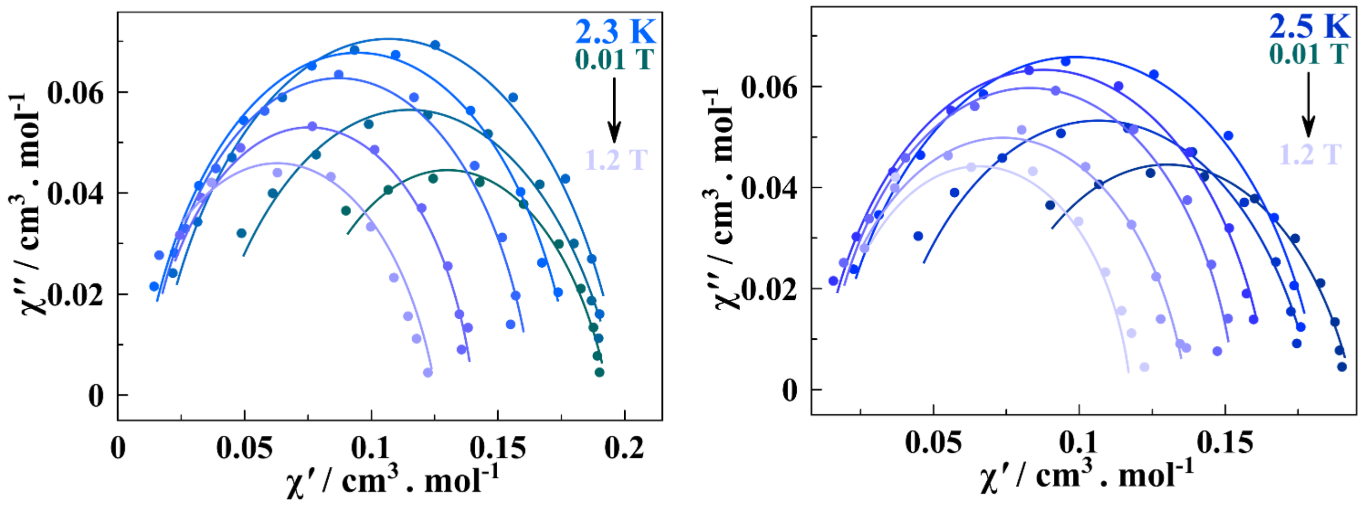

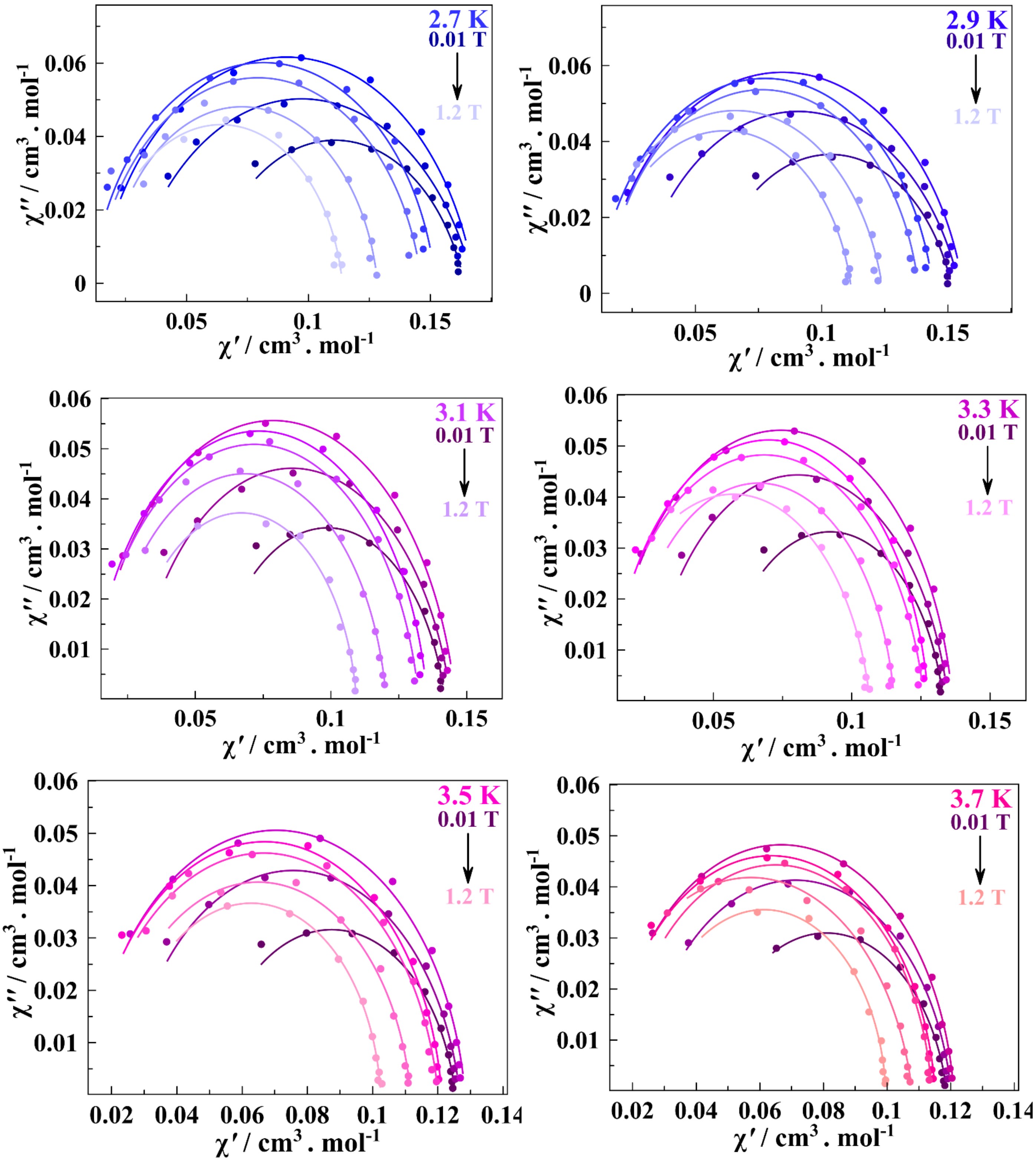
2.7 K
0.01 T
1.2 T
χ'' / cm3 . mol-1
χ' / cm3 . mol-1
2.9 K
0.01 T
1.2 T
χ'' / cm3 . mol-1
χ' / cm3 . mol-1
3.1 K
0.01 T
1.2 T
χ'' / cm3 . mol-1
χ' / cm3 . mol-1
3.3 K
0.01 T
1.2 T
χ'' / cm3 . mol-1
χ' / cm3 . mol-1
3.5 K
0.01 T
1.2 T
χ'' / cm3 . mol-1
χ' / cm3 . mol-1
3.7 K
0.01 T
1.2 T
χ'' / cm3 . mol-1
χ' / cm3 . mol-1

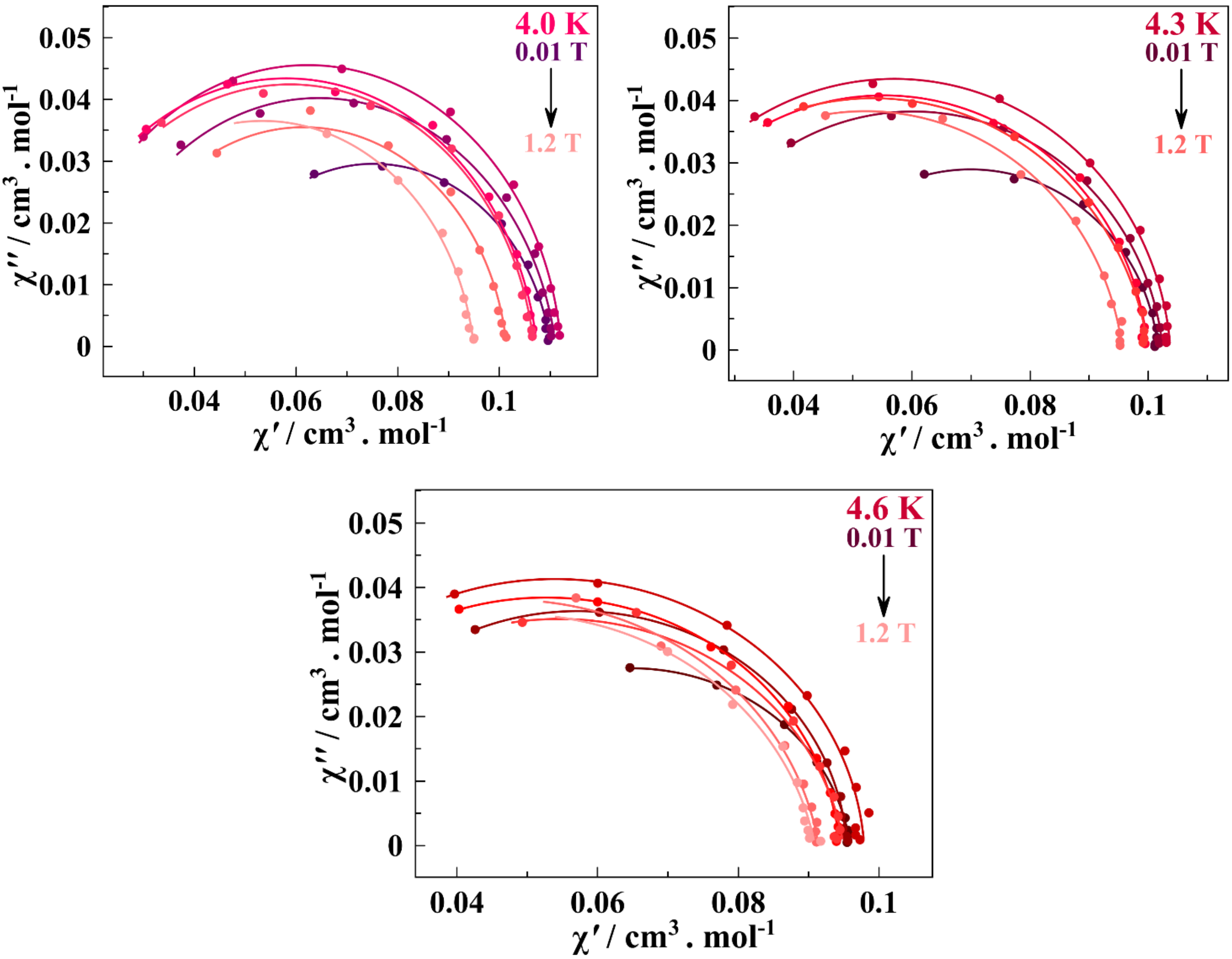

Figure S17: Cole-Cole plots recorded at different fields and fixed temperatures in the 2.3-4.6 K temperature range. Solid lines indicate the best fits.

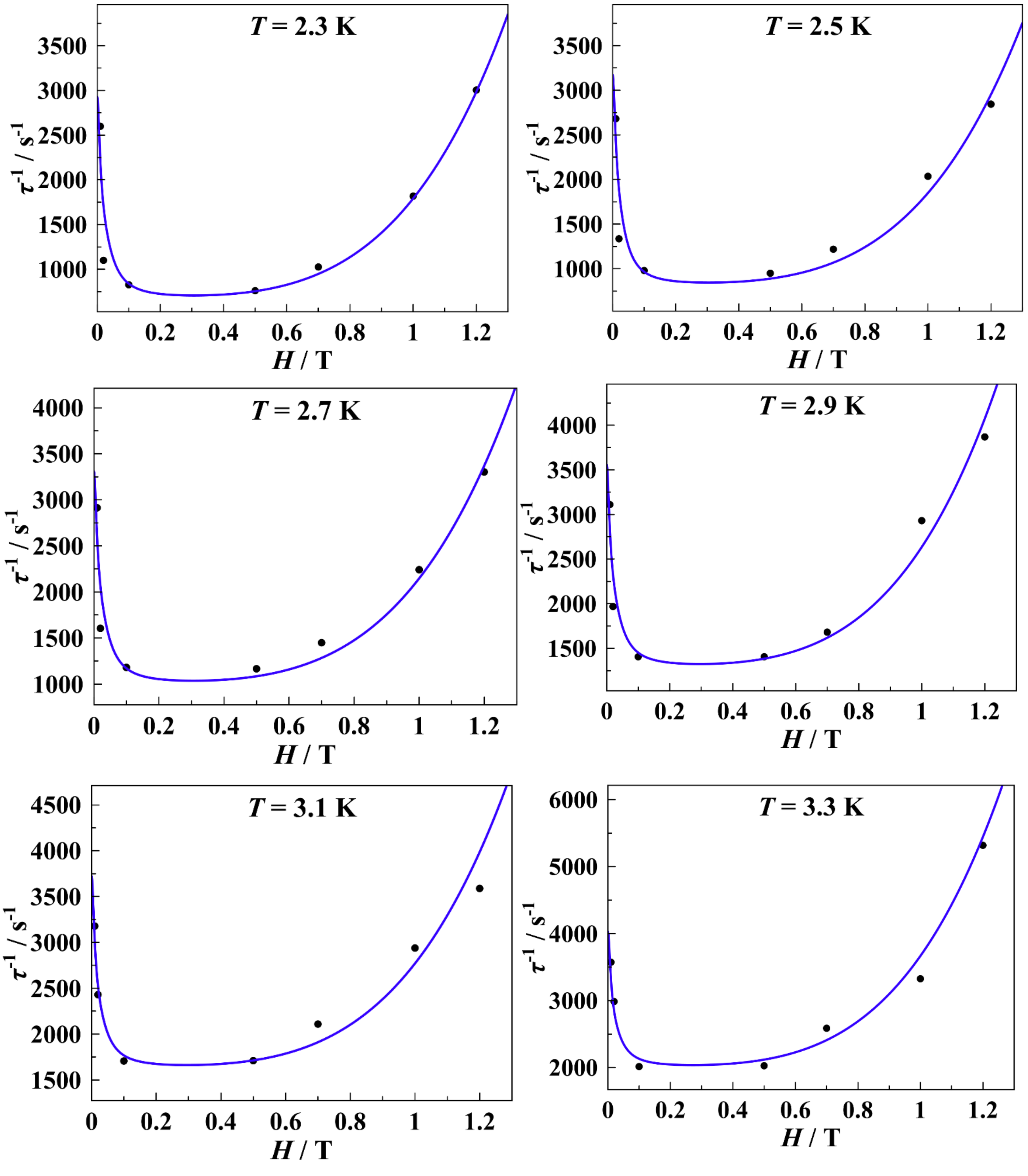
T = 2.3 K
T = 2.5 K
T = 2.7 K
T = 2.9 K
T = 3.1 K
T = 3.3 K
τ-1 / s-1
H / T

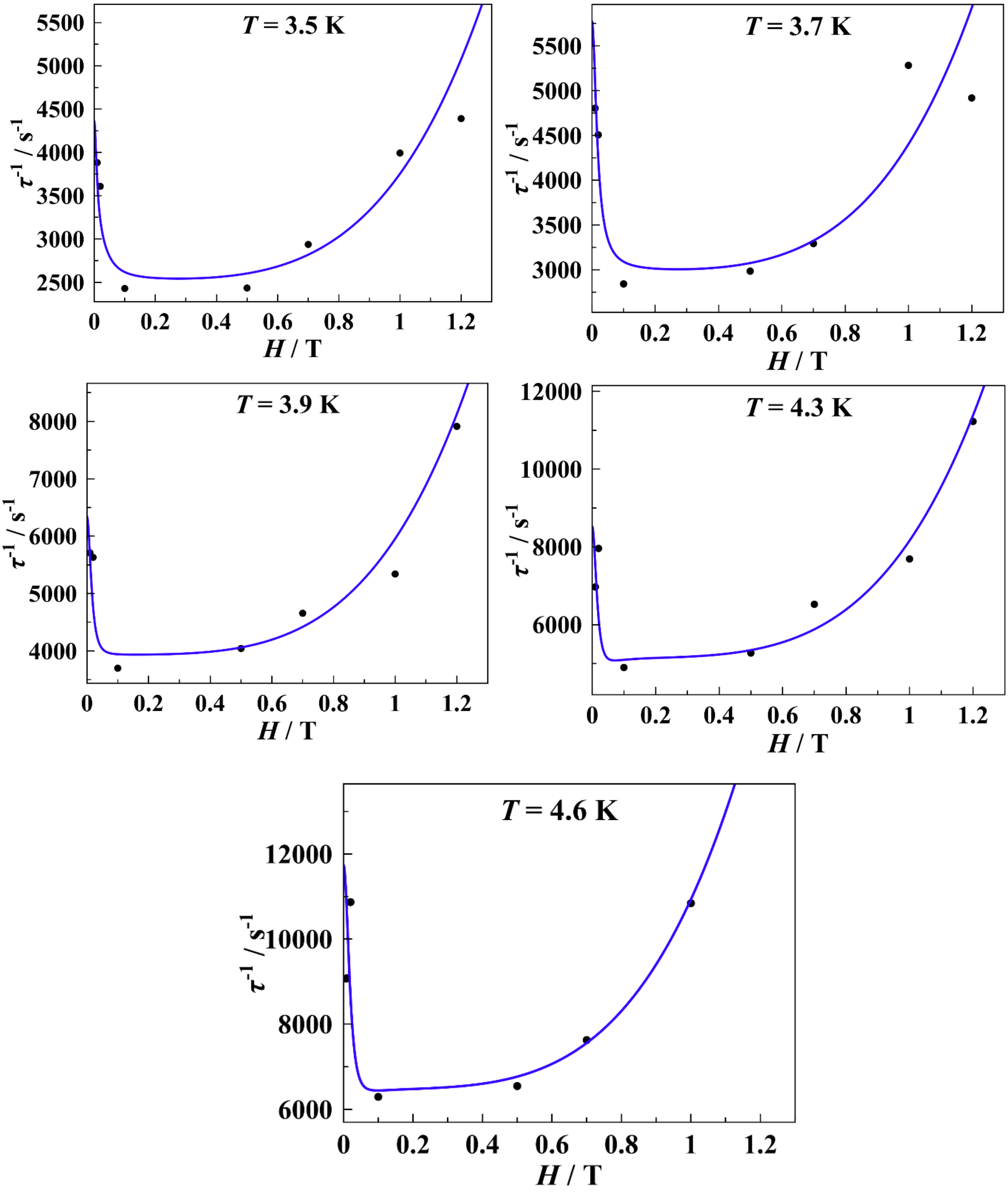

Figure S18: Relaxation rate dependence on the applied DC field recorded at different temperatures. Solid lines correspond to the best fits.

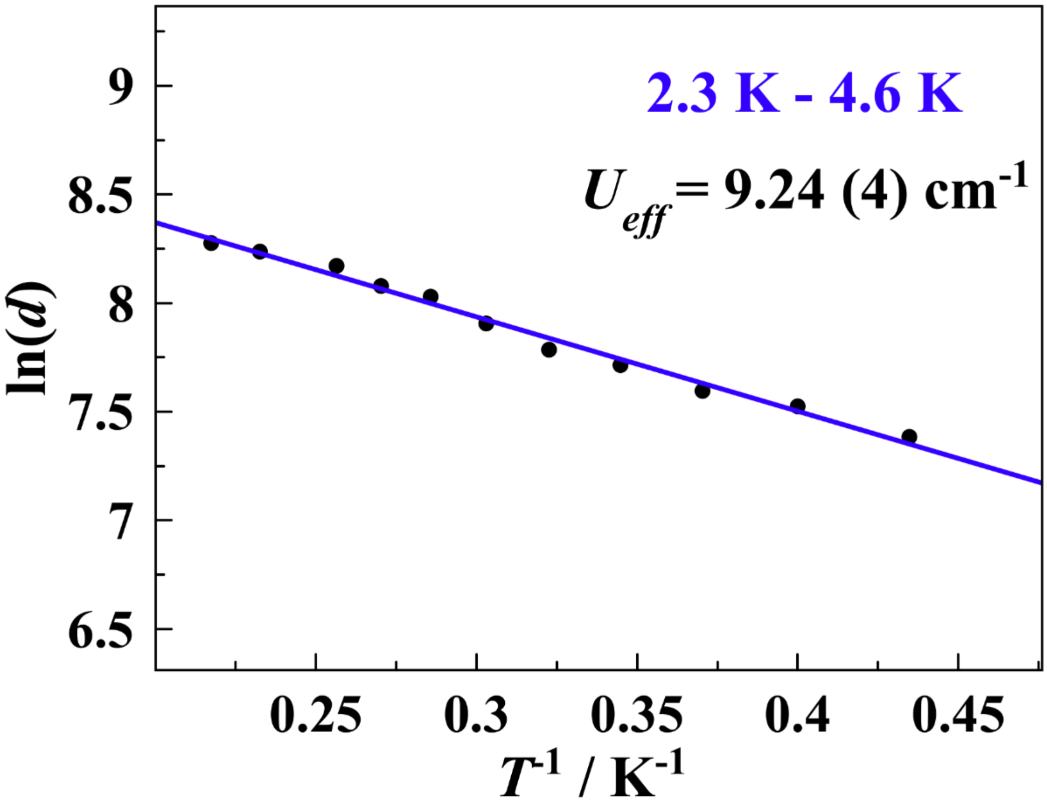

Figure S19: Arrhenius plot of the *d* parameters extracted from the Brons-van Vleck model fitting.

Table S3: Different combinations of relaxation pathways employed to fit the ac data from compounds **1** and **4** at 0.02 T.

Raman + QTM			
	C	*n*	*QT* (s)
**1 @ 0.02 T**	15(3)	4.2(1)	531(67)
**4 @0.02 T**	5.2(4)	4.52(5)	1004(17)
Local modes + QTM			
	D (s^{-1})	ω (cm^{-1})	*QT* (s)
**1 @ 0.02 T**	$5.26 \cdot 10^5$ ($1.29 \cdot 10^4$)	12.64(6)	878(42)
**4 @ 0.02 T**	$3.49 \cdot 10^5$($7.9 \cdot 10^3$)	13.46(6)	1144(26)
Exponential (specific temperature range)			
	τ_0^{-1}(s^{-1})	U_{eff} (cm^{-1})	
**1 @ 0.02 T**	$1.7 \cdot 10^{-6}$($2.2 \cdot 10^{-7}$)	9.34(1)	
**4 @ 0.02T**	$2.2 \cdot 10^{-6}$($6.9 \cdot 10^{-6}$)	8.91(4)	

Table S4. Tau vs. temperature values for compound 1 at the measured fields.

0.05 T		0.1 T	
T (K)	τ (s)	T (K)	τ (s)
2.30119	0.001491	2.30119	0.002733
2.50051	0.00124	2.50051	0.002287
2.70037	0.001039	2.70037	0.001934
2.89987	9.22E-04	2.89987	0.001666
3.10016	7.84E-04	3.10016	0.001442
3.30008	6.86E-04	3.30008	0.001263
3.4999	6.07E-04	3.4999	0.001109
3.79992	5.09E-04	3.79992	9.34E-04
4.0921	4.38E-04	4.0921	8.01E-04
4.39951	3.77E-04	4.39951	6.80E-04
4.69758	3.36E-04	4.69758	5.97E-04
4.99728	2.84E-04	4.99728	5.15E-04
5.49793	2.46E-04	5.49793	4.15E-04
5.99676	1.98E-04	5.99676	3.38E-04
6.49791	1.66E-04	6.49791	2.77E-04
6.99605	1.42E-04	6.99605	2.40E-04
7.4958	1.30E-04	7.4958	1.96E-04
7.99396	1.03E-04	7.99396	1.71E-04
8.99461	8.89E-05	8.99461	1.22E-04
9.99668	7.84E-05	9.99668	9.40E-05
10.88828	6.17E-05	10.88828	7.98E-05
12.00078	5.11E-05	12.00078	5.74E-05
		13.99801	4.92E-05
		15.99953	3.90E-05
		17.99997	3.41E-05
**0.3 T**		**0.6 T**	
**T (K)**	**τ (s)**	**T (K)**	**τ (s)**
2.30119	0.012024	2.30038	0.020067
2.50051	0.009883	2.50047	0.01557
2.70037	0.008247	2.69988	0.012601
2.89987	0.007081	2.8996	0.010762
3.10016	0.006162	3.09922	0.009286
3.30008	0.005368	3.29874	0.008079
3.4999	0.004793	3.49963	0.007185
3.79992	0.003984	3.79906	0.006061

4.0921	0.003382	4.10181	0.005202
4.39951	0.002885	4.39774	0.004496
4.69758	0.002551	4.69771	0.004053
4.99728	0.002196	5.00028	0.003595
5.49793	0.00177	5.50024	0.00308
5.99676	0.001451	5.99992	0.002621
6.49791	0.001209	6.50038	0.002306
6.99605	0.001012	7.00038	0.002021
7.4958	8.43E-04	7.50021	0.001805
7.99396	7.15E-04	8.00051	0.001633
8.99461	5.35E-04	8.99967	0.001346
9.99668	3.99E-04	9.99975	0.001115
10.88828	2.93E-04	10.88987	9.67E-04
12.00078	2.10E-04	11.99916	7.77E-04
13.99801	1.41E-04	14.0004	5.68E-04
15.99953	1.04E-04	16.00009	4.68E-04
17.99997	8.33E-05	18.00009	3.45E-04
		20.00023	2.82E-04
		22.99932	2.44E-04
		26.00016	1.73E-04
		29.99983	1.33E-04
		35.00009	1.16E-04
		39.99987	6.82E-05
**1 T**		**1.5 T**	
**T (K)**	**τ (s)**	**T (K)**	**τ (s)**
2.30067	0.07699	6.5	0.003538
2.5	0.050375	7	0.003082
2.7	0.04267	7.50031	0.002705
2.90001	0.034072	8.00028	0.002392
3.0998	0.029378	8.99996	0.001892
3.29991	0.024152	9.99993	0.00155
3.49988	0.020432	10.89123	0.001258
3.80237	0.016645	11.99953	9.84E-04
4.10094	0.013387	13.99969	7.03E-04
4.39812	0.011317	15.9999	5.20E-04
4.69965	0.00971	18.00021	4.08E-04
5.00021	0.008446	20.00038	3.32E-04
5.50095	0.007027	22.99993	2.44E-04
6.00003	0.005888	26.00068	1.80E-04
6.49989	0.005036	30.00001	1.34E-04
6.99992	0.004339	34.99961	9.39E-05
7.49986	0.003724	40.00081	6.73E-05
7.9997	0.003299		
8.99892	0.002581		

10.0004	0.002006		
10.891	0.001614		
11.9996	0.001218		
14.0003	8.38E-04		
16.0003	6.13E-04		
18.0001	4.65E-04		
20.0001	3.67E-04		
22.9999	2.67E-04		
26.0004	2.08E-04		
29.9995	1.51E-04		
35.0001	1.05E-04		
40.0005	8.09E-05		
**2 T**		**2.5 T**	
**T (K)**	**τ (s)**	**T (K)**	**τ (s)**
6.5	0.002134	6.5	0.0013
7	0.001928	7	0.001228
7.50031	0.001743	7.50031	0.001179
8.00028	0.001645	8.00028	0.00107
8.99996	0.001366	8.99996	9.40E-04
9.99993	0.001127	9.99993	8.03E-04
10.89123	9.48E-04	10.89123	6.94E-04
11.99953	8.33E-04	11.99953	6.04E-04
13.99969	5.61E-04	13.99969	4.22E-04
15.9999	4.65E-04	15.9999	3.70E-04
18.00021	3.56E-04	18.00021	3.15E-04
20.00038	2.60E-04	20.00038	2.46E-04
22.99993	1.70E-04	22.99993	1.91E-04
26.00068	1.38E-04	26.00068	1.66E-04
30.00001	1.07E-04	30.00001	1.01E-04
34.99961	8.49E-05	34.99961	7.53E-05
40.00081	6.19E-05	40.00081	6.68E-05
**3 T**			
**T (K)**	**τ (s)**		
6.5	8.53E-04		
7	7.47E-04		
7.50031	8.05E-04		
8.00028	7.09E-04		
8.99996	7.11E-04		
9.99993	5.47E-04		
10.89123	5.17E-04		
11.99953	4.11E-04		
13.99969	3.63E-04		
15.9999	2.64E-04		

18.00021	2.86E-04
20.00038	1.83E-04
22.99993	1.52E-04
26.00068	1.38E-04
30.00001	9.25E-05
40.00081	5.85E-05

Table S5: Best fitting parameters for compound **1** used to reproduce the relaxation rate dependence with temperature.

T (K)	C ($T^{-4}s^{-1}$)	d (ms^{-1})	e (T^{-2})	f (T^{-2})
**6.5**	12.2(9)	7.8(2)	3.4(6)	124(8)
**7**	13.5(8)	9.4(2)	3.5(5)	133(7)
**7.5**	12(1)	9.5(2)	2.6(5)	95(6)
**8**	14(1)	13.1(2)	3.1(4)	129(6)
**9**	16(3)	12.9(4)	1.2(7)	61(8)
**10**	22(9)	14.3(8)	0.4 (1.04)	39(11)
**10.9**	20(3)	19.9(4)	1.2(4)	55(5)
**12**	27(7)	22.3(7)	0.53(0.53)	39(5)

Table S6. Best-fit parameters of the model used to reproduce the field dependence of the magnetization relaxation for 1.

B (T)	C($s^{-1}K^{-n}$)	n	A($s^{-1}K^{-1}$)	T (K)
**0.05**	119(2)	2.09(1)	-	2.3-12
**0.1**	57.3(3)	2.198(3)	-	2.3-18
**0.3**	12.4(3)	2.31(1)	-	2.3-18
**0.6**	11.1(2)	1.928(7)	-	2.3-40
**1**	2.51(9)	2.32(2)	-	2.3-40
**1.5**	2.7(3)	2.32(4)	11(2)	6.5-40
**2**	3.2(1)	2.3(1)	30(3)	6.5-40
**2.5**	2.6(1)	2.301(1)	79(4)	6.5-40
**3**	2.3(2)	2.30(1)	138(8)	6.5-40

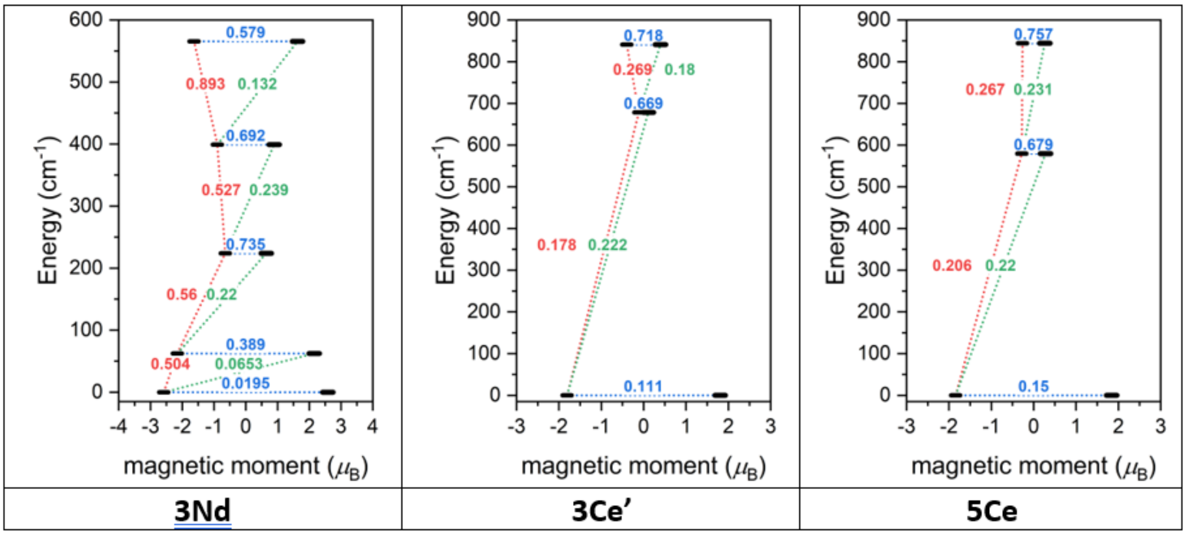

Figure S20: The CASSCF/SINGLE_ANISO calculations of ab initio magnetization blocking barriers for compounds **3Nd**, **3Ce’**, and **5Ce**. The numbers presented in the plots represent the corresponding matrix elements of the transversal magnetic moment (for values larger than 0.1 and efficient relaxation mechanism is expected).

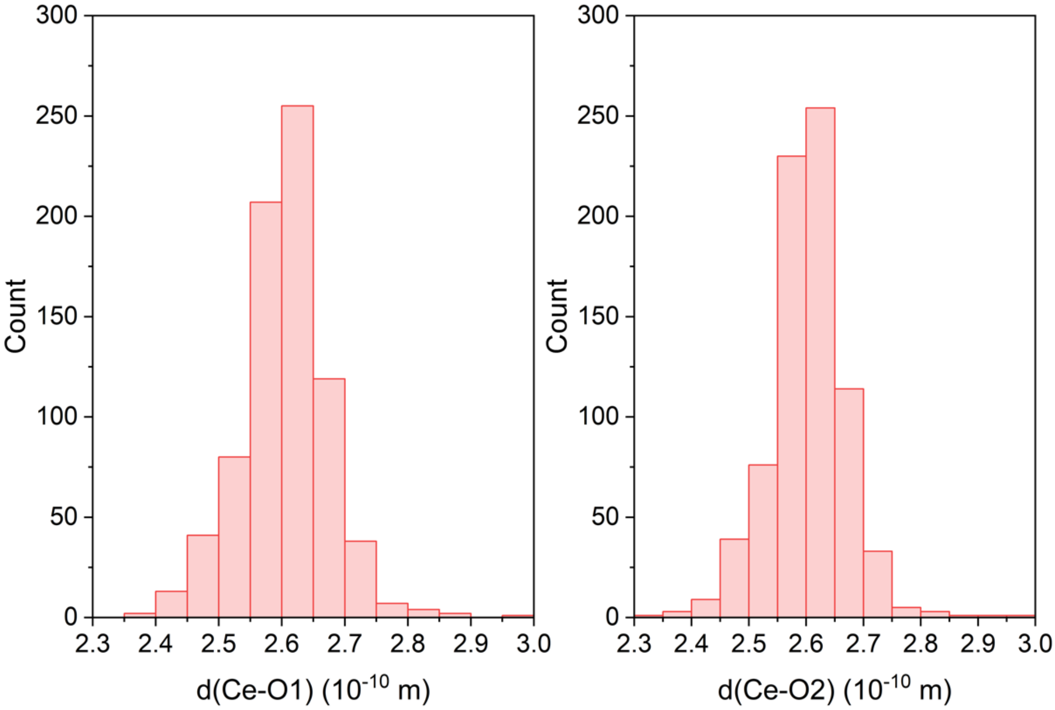

Figure S21: Histogram of the Ce-O1$_{nitrato}$ and Ce-O2$_{nitrato}$ distances of every published structure featuring Ce^{III}-nitrato coordination bonds.

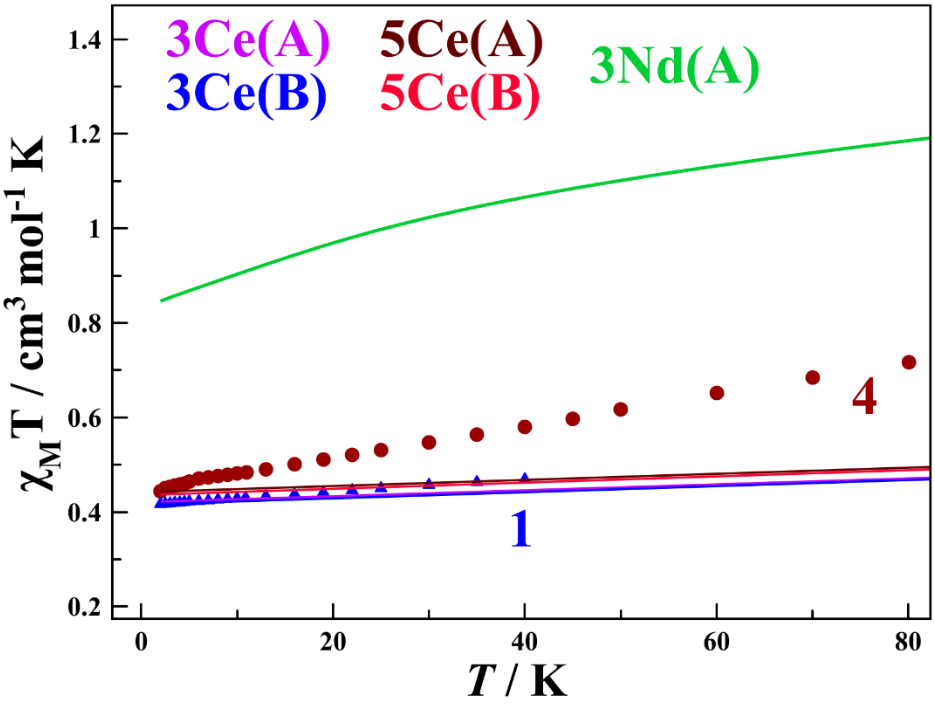

Figure S22: The magnetic properties calculated by CASSCF and CASSCF/NEVPT2 methods with the help of the SINGLE_ANISO module for **3Nd**, **3Ce'**, and **5Ce**. Comparison with the experimentally obtained values for compounds **1** and **4**, isostructural to **3Ce** and **5Ce**.

References

S1. Gao B.; Zhang Q.; Yan P.; Hou G.; Li G. Crystal engineering of salen type cerium complexes tuned by various cerium counteranions. *CrystEngComm*, **2013**, *15*, 4167-4175.
